# Coronavirus NSP14 drives internal m^7^G modification to rewire host splicing and promote viral replication

**DOI:** 10.1093/nar/gkag829

**Published:** 2026-08-21

**Authors:** Ensueño Esmeralda Sáenz Altamirano, Chien-Hsin Huang, Yueh-Lin Tsai, Tiffany J Tzeng, Natália Fagundes Borges Teruel, Yoatzin Peñaflor-Téllez, Shruti Chatterjee, Isha Pandey, Neelam Oswal, Y Grace Chen, Ching-Wen Chang, Ricardo Rajsbaum, Lok-Yin Roy Wong, Ivan R Corrêa, Jack Chun-Chieh Hsu

**Affiliations:** Center for Virus-Host-Innate Immunity, Institute for Infectious and Inflammatory Diseases, Rutgers New Jersey Medical School, Rutgers, The State University of New Jersey, Newark, NJ 07103, United States; Center for Virus-Host-Innate Immunity, Institute for Infectious and Inflammatory Diseases, Rutgers New Jersey Medical School, Rutgers, The State University of New Jersey, Newark, NJ 07103, United States; New England Biolabs Inc., Beverly, MA 01915, United States; Center for Virus-Host-Innate Immunity, Institute for Infectious and Inflammatory Diseases, Rutgers New Jersey Medical School, Rutgers, The State University of New Jersey, Newark, NJ 07103, United States; Center for Virus-Host-Innate Immunity, Institute for Infectious and Inflammatory Diseases, Rutgers New Jersey Medical School, Rutgers, The State University of New Jersey, Newark, NJ 07103, United States; Center for Virus-Host-Innate Immunity, Institute for Infectious and Inflammatory Diseases, Rutgers New Jersey Medical School, Rutgers, The State University of New Jersey, Newark, NJ 07103, United States; Center for Virus-Host-Innate Immunity, Institute for Infectious and Inflammatory Diseases, Rutgers New Jersey Medical School, Rutgers, The State University of New Jersey, Newark, NJ 07103, United States; Center for Virus-Host-Innate Immunity, Institute for Infectious and Inflammatory Diseases, Rutgers New Jersey Medical School, Rutgers, The State University of New Jersey, Newark, NJ 07103, United States; Center for Discovery and Innovation, Hackensack Meridian Health, Nutley, NJ 07110, United States; Department of Immunobiology, Yale University School of Medicine, New Haven, CT 06520, United States; Center for Discovery and Innovation, Hackensack Meridian Health, Nutley, NJ 07110, United States; Center for Virus-Host-Innate Immunity, Institute for Infectious and Inflammatory Diseases, Rutgers New Jersey Medical School, Rutgers, The State University of New Jersey, Newark, NJ 07103, United States; Department of Medicine, Rutgers New Jersey Medical School, Rutgers, The State University of New Jersey, Newark, NJ 07103, United States; Center for Virus-Host-Innate Immunity, Institute for Infectious and Inflammatory Diseases, Rutgers New Jersey Medical School, Rutgers, The State University of New Jersey, Newark, NJ 07103, United States; Department of Microbiology, Biochemistry, and Molecular Genetics, Rutgers New Jersey Medical School, Rutgers, The State University of New Jersey, Newark, NJ 07103, United States; New England Biolabs Inc., Beverly, MA 01915, United States; Center for Virus-Host-Innate Immunity, Institute for Infectious and Inflammatory Diseases, Rutgers New Jersey Medical School, Rutgers, The State University of New Jersey, Newark, NJ 07103, United States; Department of Medicine, Rutgers New Jersey Medical School, Rutgers, The State University of New Jersey, Newark, NJ 07103, United States

## Abstract

SARS-CoV-2 manipulates host gene expression through multiple mechanisms, including disruption of RNA processing. Here, we identify a novel function of the viral non-structural protein 14 (NSP14) in inducing *N*^7^-methylguanosine (m^7^G) modification in the internal sequences of host mRNA. We demonstrate that NSP14 catalyzes the conversion of GTP to m^7^GTP, which is subsequently incorporated into mRNA by RNA polymerase II, resulting in widespread internal m^7^G modification. This activity is dependent on NSP14’s *N*^7^-methyltransferase (*N*7-MTase) domain, and the NSP10–NSP14 interaction increases cellular m^7^G levels primarily by increasing NSP14 protein abundance. NSP14-induced m^7^G modification is conserved across alpha-, beta-, and gamma-coronaviruses. Mechanistically, we show that this RNA modification is associated with altered splicing, particularly in genes regulating genome stability, RNA metabolism, and nuclear processes. Importantly, using SARS-CoV-2 infection models, we show that viral replication is associated with increased cellular m^7^G signal, supporting the relevance of this pathway during infection. Inhibition of NSP14 *N7*-MTase or RNA polymerase II reduces SARS-CoV-2 replication, consistent with a model in which NSP14-induced m^7^G modification may contribute to viral replication. Our findings reveal a previously unrecognized epitranscriptomic mechanism and suggest that NSP14-induced m^7^G modification may contribute to the remodeling of host gene expression during coronavirus infection.

## Graphical Abstract

**Graphical Abstract:**
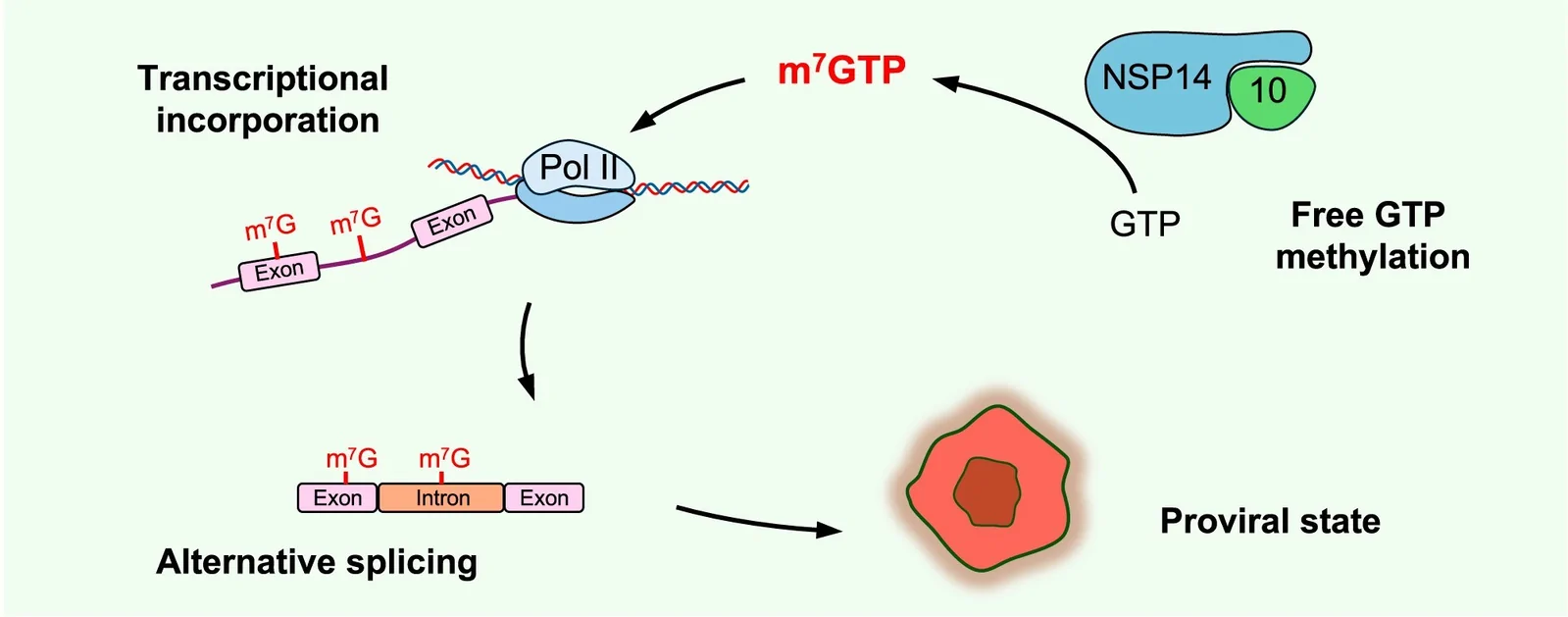

## Introduction

The COVID-19 pandemic, caused by severe acute respiratory syndrome coronavirus 2 (SARS-CoV-2), emerged in late 2019 and continues to pose a significant global health challenge. As a member of the *Coronaviridae* family, SARS-CoV-2 contains a positive-sense, single-stranded RNA genome of ∼30 kb [1]. The viral genome is enclosed by a 5′ cap and a 3′ poly(A) tail, allowing it to function as mRNA for viral protein synthesis [2]. The 5′ end of the genome encodes two large open reading frames (ORF1a and ORF1ab), which are translated into polyproteins pp1a and pp1ab. These polyproteins are subsequently processed into 16 non-structural proteins (NSP1–NSP16) [3]. Each NSP plays a distinct role in viral replication and transcription, contributing to the formation of the replicase–transcriptase complex (RTC) [4–6]. Moreover, NSPs also play important roles in manipulating host gene expression and immune responses, contributing to viral replication and immune evasion [3, 7].

RNA modifications are chemical alterations to RNA molecules that play crucial roles in gene expression and RNA metabolism. To date, >170 distinct types of RNA modifications have been identified [8]. Among these, *N*^7^-methylguanosine (m^7^G) is one of the most prevalent modifications [9]. Although m^7^G is known for its presence at the 5′ cap of mRNA, it also occurs within the internal sequences of various RNA species, including rRNA, tRNA, mRNA, and non-coding RNA [10]. These modifications are catalyzed by a group of *N*^7^-methyltransferases (*N7*-MTases), also known as “m^7^G writers”, which methylate the guanine nucleobase at the *N*^7^ position, forming m^7^G [10]. During transcription, mRNA undergoes co-transcriptional m^7^G modification at the 5′ cap by the enzyme RNA guanine-7 methyltransferase (RNMT), which is essential for RNA stability, splicing, nuclear export, and translation initiation [11, 12]. In addition to capping, m^7^G modification is found in internal sequences. In tRNA, m^7^G at position G46 is installed by the METTL1–WDR4 complex and plays a critical role in RNA tertiary structure stabilization and translation fidelity [13, 14]. In 18S rRNA, m^7^G at position G1639 is modified by the WBSCR22–TRM112 complex and supports ribosome biogenesis and translation initiation [15, 16]. Recent studies revealed the presence of m^7^G modifications in the internal sequences of mRNA [17]. Internal m^7^G/G levels range between 0.02% and 0.05%, corresponding to ∼800 sites across mammalian transcriptomes [17]. These internal m^7^G modifications, catalyzed by the METTL1–WDR4 complex, play important roles in translational regulation and stress responses [17–20].

RNA modifications are prevalent strategies used by viruses to regulate both host and viral gene expression, crucial in controlling viral replication and evading host immunity [21]. Coronaviruses encode two RNA methyltransferases, NSP14 and NSP16, that modify the viral RNA 5′ cap to form a functional cap structure that mimics the host mRNA cap. The 5′ cap plays a critical role in viral RNA translation and immune evasion of type I interferon (IFN-I) response [22]. NSP14 is a bifunctional enzyme with distinct *N*7-MTase and exoribonuclease (ExoN) domains [23, 24]. The *N*7-MTase activity methylates the viral 5′ cap guanosine into m^7^G, forming a Cap-0 structure [25]. In contrast, the NSP10–NSP16 complex functions as a 2′-*O*-methyltransferase (2′-*O*-MTase), specifically catalyzing methylation at the 2′-*O*-ribose of the first nucleotide of the viral RNA [25]. Beyond the viral RNA capping activity, SARS-CoV-2 NSP14 plays a critical role in host RNA metabolism at the post-transcriptional level. NSP14 inhibits host translation, resulting in a reduction in IFN-I-dependent interferon-stimulated gene (ISG) induction at the protein level [26]. In addition, NSP14 inhibits mRNA export from the nucleus to the cytoplasm and alters host mRNA splicing patterns [27, 28]. Notably, the *N*7-MTase activity is essential for SARS-CoV-2 replication. Recombinant SARS-CoV-2 harboring *N*7-MTase inactive mutations exhibits a significant attenuation in viral replication [29]. Moreover, inhibition of *N*7-MTase by small-molecule inhibitors restricts viral replication both *in vitro* and *in vivo* [30, 31].

In this study, we investigate the role of SARS-CoV-2 NSP14 in m^7^G modification of cellular RNA. We report that NSP14 overexpression leads to increased internal m^7^G modification in mRNA. Importantly, SARS-CoV-2 infection and viral replicon particle experiments show increased cellular m^7^G signal in host cells, supporting the relevance of this pathway during viral replication. Using *N*7-MTase-inactive mutants and *N*7-MTase inhibitors, we demonstrate that the *N*7-MTase activity is essential for this modification. Moreover, the NSP14–NSP10 interaction increases NSP14 protein abundance and enhances cellular m^7^G levels. Notably, this m^7^G modification activity is conserved across alpha-, beta-, and gamma-coronaviruses, including human coronaviruses, murine hepatitis virus (MHV), and avian infectious bronchitis virus (IBV). In addition, NSP14 induces m^7^G modification through transcriptional incorporation by hijacking cellular RNA polymerases (Pols). Specifically, NSP14 catalyzes the conversion of GTP into m^7^GTP, which is then incorporated into mRNA by RNA Pol II. Moreover, NSP14-induced m^7^G modification is associated with widespread changes in host mRNA splicing, including increased intron retention, suggesting that this modification may affect host RNA processing and antiviral gene expression programs relevant to viral replication. Consistent with this model, virus infection-based inhibitor experiments further suggest that NSP14 *N7*-MTase activity and RNA Pol II-dependent m^7^G incorporation contribute to SARS-CoV-2 replication. Our results provide insights into a mechanism by which coronavirus NSP14 rewires host RNA modification and splicing programs, with potential consequences for viral replication.

## Materials and methods

### Plasmids

Viral protein constructs were generated as described previously [26]. DcpS, a scavenger decapping enzyme [32], was cloned from HEK293T cDNA into the pCAGGS-FLAG vector. NSP10, NSP14, and DcpS mutants were generated using site-directed mutagenesis. All primers used for molecular cloning and quantitative reverse transcription–polymerase chain reaction (qRT–PCR) were synthesized by Integrated DNA Technologies (IDT). Plasmids were purified using Zymo Research kits.

### Cell culture

HEK293T, HeLa, Huh7, and Vero E6 cells were cultured in Dulbecco’s modified Eagle’s medium (DMEM; Corning) supplemented with 10% fetal bovine serum (FBS; Corning). For DNA plasmid and mRNA transfection, cells were plated 24 h before transfection and transfected using Lipofectamine 2000 according to the manufacturer’s instructions. For inhibitor experiments, cells were pre-treated with the indicated concentrations of inhibitors for 24 h prior to transfection or infection. Inhibitors remained in the culture medium throughout the post-transfection or post-infection period unless otherwise specified.

### Viruses, virus replicon particle, and cell culture infections

The SARS-CoV-2 virus (isolate USA-WA1/2020) was obtained from the BEI Resources repository. All infection experiments were conducted under biosafety level 3 (BSL-3) containment as described previously [33, 34]. Vero E6 cells were mock-infected or infected with the virus at the indicated multiplicity of infection (MOI) in serum-free DMEM for 1 h. For virus titering, Vero E6 cells were incubated with the inoculum for 1 h. After removing inoculum, plates were overlaid with 1.2% methylcellulose containing 2% FBS for 24 h. Cells were fixed with 4% paraformaldehyde (PFA) or 10% formaldehyde for 1 h at room temperature, washed three times with phosphate-buffered saline (PBS), and stained with anti-nucleocapsid antibody for SARS-CoV-2 (1:1000) for 1 h at 37 °C. Cells were then washed three times with PBS and stained with horseradish peroxidase (HRP)-conjugated secondary antibody for 1 h at 37 °C. Foci were visualized by peroxidase substrate, after washing the cells twice with PBS. Viral titers were quantified as fluorescent-focus units (FFU)/ml.

The SARS-CoV-2 isolate XEC (V7447) was isolated from a nasopharyngeal clinical sample at the Hackensack Meridian Health Center for Discovery and Innovation (HMH-CDI). Viruses were propagated in Vero E6-Tmprss2 cells in a growth medium consisting of DMEM and 2% heat-inactivated FBS. Virus infections were performed in A549-Ace2-Dpp4-Tmprss2 (ADT) cells for 24 h at 37°C in infection medium (DMEM, 10% FBS) [35]. Immunofluorescence-based viral replication assay and cytotoxic assay were performed as described previously [35]. In brief, immunofluorescence staining for viral nucleocapsid protein (NP) was performed to determine viral replication activity. Infected cells were fixed in 4% PFA, washed with PBS, permeabilized with Triton X-100 (0.1%)/PBS, and blocked with blocking solution [0.1% Triton X-100 and 0.2% bovine serum albumin (BSA) in PBS]. Cells were stained with NP-specific antibodies, Alexa-594-conjugated secondary antibodies, and 4′,6-diamidino-2-phenylindole (DAPI). Images were acquired with Cytation C10 and processed using the Gen 5 Image Software (Agilent BioTek). The toxicity of the compounds was measured using CellTiter Glo with a Tecan Infinite M200PRO plate reader to measure the cell viability by luminescence.

For virus replicon particle (VRP) experiments, the single-cycle infectious SARS-CoV-2 replicon system was generated in Huh7 cells as described previously [36]. For VRP exposure, Huh7 cells were incubated with VRP-containing supernatant for 24 h for immunofluorescence analysis.

### Protein immunoblotting

Cells were lysed in Laemmli buffer and boiled for 10 min. Cell lysates were separated by sodium dodecyl sulfate–polyacrylamide gel electrophoresis (SDS–PAGE) with 4–15% Mini-PROTEAN TGX gels (Bio-Rad) and transferred onto polyvinylidene fluoride (PVDF) membranes (Millipore). Membranes were blocked in 5% BSA/Tris-buffered saline (TBS) with 0.1% Tween-20 (TBST) for 1 h at room temperature, incubated with primary antibodies in 2% BSA/TBST overnight at 4°C, followed by incubation with HRP-conjugated antibodies in 2% BSA/TBST for 1 h at room temperature. Signal detection was performed using the SuperSignal West Pico PLUS kit (Thermo Scientific) in a ChemiDoc MP Imaging System (Bio-Rad).

### RNA extraction, poly(A) RNA purification, and decapping reaction

Cells were harvested and washed with Dulbecco’s PBS (DPBS) buffer. Total RNA was extracted using TRIzol reagent (Thermo Scientific) and subsequently purified with Direct-zol RNA MiniPrep (Zymo Research). mRNA was enriched by two rounds of poly(A) purification using oligo(dT)_25_ magnetic beads (New England Biolabs). RNA concentrations were measured using a Nanodrop spectrophotometer. For the mRNA decapping reaction, poly(A) RNA was treated with mRNA Decapping Enzyme (New England Biolabs), which removes the 5′ cap from intact mRNA and is distinct from DcpS scavenger decapping, according to the manufacturer’s protocol, and purified using Oligo Clean & Concentrator (Zymo Research).

### RNA immunoblotting (m^7^G dot blot)

RNA immunoblotting was performed as described previously with minor modifications [17]. In brief, RNA was serially diluted and spotted on a nylon membrane, followed by UV-cross-linking. Membranes were blocked with 5% BSA/TBST for 1 h at room temperature and incubated with anti-m^7^G antibody (RN017M, MBL) in 2% BSA/TBST. After three washes with 1× TBST, membranes were incubated with HRP-conjugated secondary antibodies in 0.05% non-fat milk/TBST for 1 h at room temperature. Signal detection was performed using the SuperSignal West Pico PLUS kit (Thermo Scientific) in a ChemiDoc MP Imaging System (Bio-Rad).

### Confocal immunofluorescence microscopy

For infection and VRP experiments, Vero E6 cells were infected at an MOI of 0.01 and incubated for 48 h before processing. For VRP exposure experiments, Huh7 cells were incubated with VRP-containing supernatant for 24 h prior to immunofluorescence analysis. Cells were fixed with 3.7% PFA in PBS for 20 min at room temperature, followed by permeabilization with 0.5% Triton X-100 in PBS for 5 min at room temperature. Non-specific binding was blocked with 0.5% (w/v) gelatin in PBS. Cells were then incubated overnight at 4°C with primary antibodies diluted in blocking solution, including anti-m7G (1:200) and anti-NSP14 (1:100). After washing with PBS, cells were incubated with Alexa Fluor-conjugated secondary antibodies for 2 h at room temperature. Nuclei were stained with DAPI (1 μg ml^−1^) for 5 min. Slides were mounted, imaged using a Leica confocal microscope, and analyzed with ImageJ.

For the green fluorescent protein (GFP)-tagged NSP14 experiment, HeLa cells were seeded onto glass coverslips and transfected. After 24 h, the cells were washed with PBS and fixed with 4% PFA/PBS for 10 min at room temperature, followed by incubation in cold methanol for 10 min on ice. Cells were then incubated with 70% ethanol for 10 min, followed by 1 M Tris–HCl (pH 8) for 5 min. Permeabilization and blocking were performed with 1% BSA in 2× SSC buffer containing 0.1% Triton X-100 for 30 min. Anti-m^7^G antibody (1:200) in blocking buffer was applied overnight at 4°C. After washing three times with 2× SSC buffer, cells were incubated with anti-mouse IgG Fab2 (Thermo Fisher, A21237, 1:100) for 1 h at room temperature in blocking buffer. Following three washes with 2× SSC buffer, cells were stained with Hoechst 1:1 000 in 2× SSC buffer for 10 min and washed twice with 2× SSC buffer. Coverslips were mounted using ProLong Gold Anti-Fade Reagent (Thermo Fisher), and imaging was conducted using a Leica SP8 confocal microscope. Images were analyzed using ImageJ. Because free soluble small molecules are not reliably retained by standard fixation/permeabilization, antibody-based imaging was interpreted as a cellular readout of retained m^7^G-associated signal rather than as a direct measurement of free m^7^GTP.

### Real-time PCR and quantitative reverse transcription–PCR

RNA was extracted from cells using TRIzol and reverse-transcribed into cDNA. For qRT–PCR, RNA was quantified using the Luna Universal One-Step RT-qPCR Kit (New England Biolabs). Analyses were performed using an Mx3000P real-time PCR system (Stratagene). For RT-PCR, splicing patterns were analyzed using primers targeting intron–exon boundaries from the Luna Universal One-Step RT-qPCR Kit (New England Biolabs). DNA products were analyzed by 12% TBE PAGE. For experiments involving transcriptional inhibitors, total RNA was extracted using TRIzol reagent and reverse-transcribed into cDNA. Quantification of RNA levels was performed using Luna® Universal qPCR Master Mix (M3003, New England Biolabs) on a QuantStudio 3 real-time PCR system (Thermo Fisher Scientific). The following primer pairs were used: Myc CAGGACTGTATGTGGAGCGG (forward) and GTCGTTGAGAGGGTAGGGGA (reverse); U6 CGCTTCGGCAGCACATATAC (forward) and AAAATATGGAACGCTTCACGA (reverse); and 47S GCTGACACGCTGTCCTCTG (forward) and ACGCGCGAGAGAACAGCAG (reverse).

### Flow cytometry

HEK293T cells were stained with Live/Dead Fixable Near-IR Dead Cell Stain for 30 min at 4°C. Following staining, cells were washed once with PBS and centrifuged at 500 *g* for 5 min. Cells were then fixed using the Foxp3 Transcription Factor Staining Buffer Kit for 1 h at room temperature. After fixation, cells were washed twice with permeabilization buffer and centrifuged at 1200 *g* for 5 min. The cells were incubated with anti-m^7^G antibody (1:1000) and anti-HA antibody (1:5000) at room temperature for 30 min. After two washes with permeabilization buffer and centrifugation at 1200 *g* for 5 min, cells were stained with the secondary antibodies at room temperature for 30 min. Cells were washed as previously described and analyzed by flow cytometry. As with immunofluorescence, this fixed-cell assay was used to measure retained m^7^G-associated signal and was not used to quantify the soluble free m^7^GTP pool.

### *In vitro* methylation assay

*In vitro* methylation assays were performed using SAM510™:SAM Methyltransferase Assay (G-Biosciences) according to the manufacturer’s instructions. Recombinant NSP14–NSP10 protein complex was incubated with GTP, ATP, UTP, or CTP in the presence of *S*-adenosylmethionine (SAM) at 37°C. Reaction products were subsequently analyzed via liquid chromatography–quadrupole time-of-flight (LC-QToF) mass spectrometry to confirm the formation of m^7^GTP.

### Mass spectrometry

A 100–200 ng aliquot of total RNA or decapped poly(A) RNA from NSP14-transfected HEK293T cells was digested to nucleosides at 37°C overnight using a Nucleoside Digestion Mix (New England Biolabs). The digested RNAs were subsequently injected without prior purification on an Agilent 1290 Infinity II UHPLC equipped with a G7117 diode array detector and an Agilent 6495C triple-quadrupole mass spectrometer operating in positive electrospray ionization (+ESI) mode. UHPLC was conducted on a Waters XSelect HSS T3 XP column (2.1 × 100 mm, 2.5 µm) containing methanol and 10 mM ammonium acetate (pH 4.5) gradient mobile phase. Mass spectrometric data were acquired using dynamic multiple reaction monitoring (DMRM) mode. Each nucleoside species was identified based on the associated retention time and mass transition in the extracted chromatogram. Differentiation of isomeric nucleosides of methylated adenosine, guanosine, and cytidine were described in previous publications [37, 38]. For nucleoside quantification, calibration curves were constructed based on the mass responses of nucleoside standards with known concentrations, as determined by a UV spectrometer (Thermo Fisher Scientific, Evolution 220). Relative abundances were calculated by normalizing the amount of modified nucleoside to the corresponding unmodified nucleoside (e.g., m^7^G/G).

### RNA sequencing analysis

RNA-seq libraries were prepared from poly(A)-enriched or m^7^G-MeRIP-immunoprecipitated RNA using the NEBNext Ultra II RNA Library Prep Kit (New England Biolabs). Libraries were sequenced on an Illumina NovaSeq platform. Reads were aligned to the human genome (GRCh38) using STAR, and splicing events were quantified using rMATS and RSeQC. Intron retention and splice junction usage were compared across conditions.

### Alternative splicing, intron retention quantification, and Gene Ontology analysis

Raw reads were adapter-trimmed with cutadapt v4.6 (minimum length 16 nt) to remove library-specific adapters. Splicing was quantified with VAST-TOOLS v2.5.1 against VastDB hg38 (Hs2). For alternative splicing (AS), PSI was computed per replicate, and ΔPSI was defined as the difference between condition means. For intron retention, PIR/PSI was computed per replicate from VAST-TOOLS .IR2 files; replicates were retained if they met a VAST coverage rule (I5 ≥ 10 and I3 ≥ 5) or (I5 ≥ 5 and I3 ≥ 10), and EE ≥ 10, requiring ≥ 2/3 replicates with coverage per condition before computing condition means. Events were called differential when |ΔPSI| ≥ 15 and when replicate distributions showed no overlap ≥ 5 percentage points. Gene Ontology (GO) enrichment analysis was performed in R (v4.4.0) using clusterProfiler (v4.12.6). The input was the list of deduplicated genes with increased intron retention from VAST-TOOLS. Enrichment was run with enrichGO against org.Hs.eg.db (v3.19.1), using the full OrgDb annotation as background. Significance was defined as Benjamini—Hochberg-adjusted *P*-value ≤ 0.05.

### Computational evaluations

Computational analyses were based on the complex structures with PDB IDs 5C8S and 7QIF [39, 40], representing different NSP14 conformational contexts with and without NSP10. A SARS-CoV-2 NSP14 homolog model based on the 5C8S structure was generated using Modeller, a widely used tool for homology modeling [41]. To perform ligand docking, GetCleft was first used to identify the binding cavity based on the original ligand positions. Subsequently, FlexAID was employed to dock the evaluated compounds into the defined cavity [42, 43]. Both docking tools were used as implemented in NRGSuite-Qt [44]. Docking calculations were performed in five independent replicates using a 500 × 500 search configuration, and the top five poses from each run, ranked by the FlexAID scoring function, were selected for analysis. Additional docking was performed using the 5C8S homolog structure, with SAM explicitly included and kept fixed at its position in the 5C8T experimental complex. To assess binding interactions, Surfaces was used to calculate per-residue contributions to the binding affinity between NSP14 and the original ligands GpppA and m^7^GpppG, as well as the docked compounds [45]. Structural visualizations were generated using PyMOL.

### Statistical analyses

Data are presented as the mean ± standard deviation (SD). Statistical comparisons between groups were made using a two-tailed unpaired Student’s *t*-test. A *P*-value < 0.05 was considered statistically significant. Statistical analyses were performed using GraphPad Prism 10.

## Results

### NSP14 induces internal m^7^G modification in mRNA

**Figure 1.**
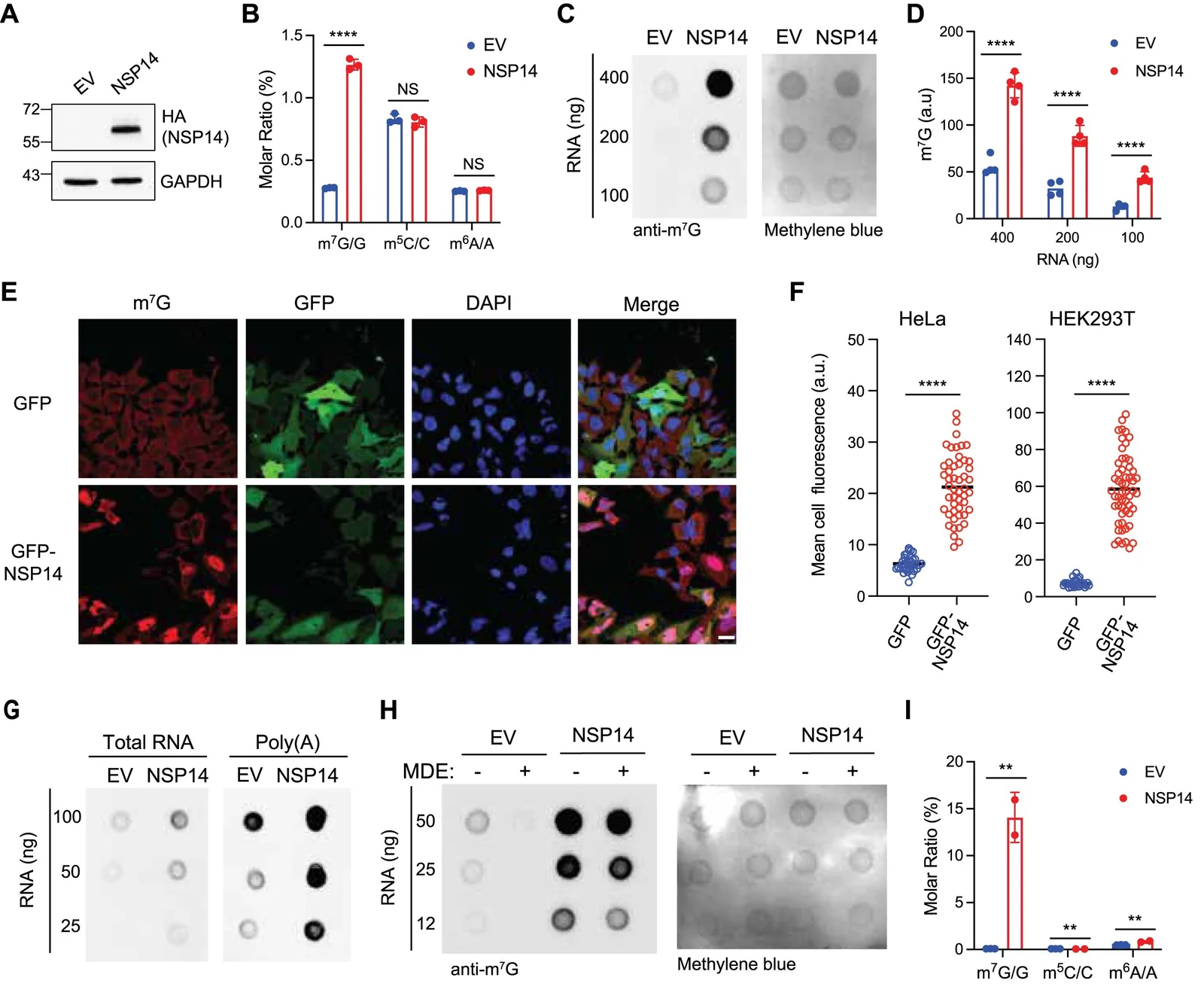
NSP14 induces internal m^7^G modification. HEK293T cells were transfected with plasmids encoding HA-tagged NSP14 or an empty vector (EV) for 24 h. (**A**) Immunoblot analysis of HA-NSP14 expression in whole-cell lysates using anti-HA and anti-GAPDH antibodies. (**B**) LC-MS/MS analysis of total RNA extracted from HEK293T cells transfected with EV or NSP14. RNA was extracted with TRIzol, digested into nucleosides, and analyzed by LC-MS/MS. The results represent the molar ratio of modified to unmodified nucleosides. (**C**) RNA m^7^G immunoblot analysis of total RNA from EV- or NSP14-transfected cells. Total RNA was extracted with TRIzol and subjected to m^7^G immunoblotting. Methylene blue staining was used as a loading control. The intensity of m^7^G signals from four biological replicates is quantified in (**D**). a.u., arbitrary unit. (**E**) Immunofluorescence analysis of HeLa cells transfected with plasmids encoding GFP or GFP-tagged NSP14. After 24 h of transfection, cells were fixed and stained with anti-m^7^G antibody. The GFP signal was used to identify transfected cells. Scale bar, 25 µm. (**F**) Quantification of m^7^G fluorescence intensity from individual HeLa and HEK293T cells. (**G**) RNA m^7^G immunoblot analysis of poly(A)-selected RNA from EV- or NSP14-transfected cells. RNA was extracted with TRIzol, isolated using oligo(dT), and subjected to m^7^G immunoblotting. (**H**) RNA m^7^G immunoblot analysis of decapped, poly(A)-selected RNA from EV- or NSP14-transfected cells. RNA was extracted with TRIzol, isolated using oligo(dT), decapped using mRNA decapping enzyme (MDE), and subjected to m^7^G immunoblotting. (**I**) LC-MS/MS analysis of decapped, poly(A)-selected RNA from EV- or NSP14-transfected cells. RNA was extracted with TRIzol, isolated using oligo(dT), decapped with MDE, digested into nucleosides, and analyzed by LC-MS/MS. The results represent the molar ratio of modified to unmodified nucleosides. For (B), (D), (F), and (I), data are shown as the mean ± SD of 2–4 biological replicates. **P < 0.01, ****P < 0.001 by unpaired Student’s *t*-test. NS, not significant.

Functional screenings in yeast revealed that SARS-CoV NSP14 can complement the loss of mRNA m^7^G capping activity [46], suggesting that NSP14 might modify cellular RNA. Given the high sequence and structural homology of NSP14 proteins between SARS-CoV and SARS-CoV-2 [26], we hypothesized that SARS-CoV-2 NSP14 can also modify host RNA. To test this, the levels of RNA modification on cellular RNA were assessed by transfecting HEK293T cells with a plasmid encoding SARS-CoV-2 NSP14. Total RNA was extracted using TRIzol and analyzed by LC-tandem mass spectrometry (LC-MS/MS) to determine the levels of three RNA methylations (m^7^G, m^5^C, and m^6^A). NSP14 expression was determined by immunoblot analysis (Fig. 1A). The results showed endogenous RNA m^7^G, m^5^C, and m^6^A modifications in the cells transfected with an empty vector control (Fig. 1B). The m^6^A level was consistent with previously reported ranges [47], whereas the levels of m^7^G and m^5^C have not been previously determined. We found that overexpression of NSP14 significantly increased m^7^G modification by 4.6-fold, whereas m^6^A and m^5^C levels remained unchanged (Fig. 1B), suggesting that NSP14 induces m^7^G modification. To validate the increase in m^7^G, we performed immunoblot analysis using an m^7^G antibody, as described previously [17]. Consistent with the LC-MS/MS results, immunoblotting revealed a significant increase in m^7^G levels upon NSP14 overexpression (Fig. 1C, D).

We next examined whether NSP14-induced m^7^G could also be detected at the cellular level using confocal immunofluorescence microscopy. HeLa cells were transfected with plasmids encoding GFP or GFP-tagged NSP14 and stained with anti-m^7^G antibody. Cells expressing GFP-tagged NSP14 showed increased m^7^G signal compared with control GFP-expressing cells, suggesting that NSP14 expression is sufficient to increase cellular m^7^G signal (Fig. 1E, F). Since m^7^G immunofluorescence relies on anti-m^7^G staining in fixed cells, we interpret this signal as retained cell-associated m^7^G rather than as detection of free m^7^G-containing metabolites, such as m^7^GTP. Standard fixation/permeabilization is expected to poorly retain free small metabolites [49], whereas RNA-associated m^7^G species are more likely to remain detectable. Similarly, RNA dot blotting is performed on purified RNA, in which free nucleotides, such as m^7^GTP, are removed during extraction and purification.

To determine if NSP14 induces m^7^G modification in mRNA, poly(A) RNA was isolated from total RNA using oligo(dT) purification and analyzed by immunoblotting. The results showed a significant increase in m^7^G in poly(A) RNA upon NSP14 expression (Fig. 1G), suggesting that NSP14 induces m^7^G in mRNA. Since mature mRNA is m^7^G-modified at the 5′ cap, we next evaluated whether NSP14-induced m^7^G can occur in internal sequences. To test this, we removed the 5′ m^7^G cap using mRNA decapping enzyme (MDE), which hydrolyzes the pyrophosphate bonds between α and β phosphate moieties within the 5′–5′ triphosphate linkage, producing a 5′ monophosphate end [48]. Using capped *in vitro* transcribed RNA as a positive control, we confirmed that MDE efficiently removed the cap-derived m^7^G signal (Supplementary Fig. S1). The results demonstrated that MDE treatment significantly reduced the m^7^G levels in the control, whereas the NSP14-induced m^7^G signal remained robust (Fig. 1H). These results suggest that NSP14 induces m^7^G in internal sequences. To validate the internal modification, the oligo(dT)-isolated, MDE-decapped RNA was subjected to LC-MS/MS analysis. Previous studies demonstrated an endogenous m^7^G to G ratio between 0.02% and 0.05% in cellular mRNA from various mammalian cells [17]. Consistently, in the control cells, we observed a basal level of m^7^G (Fig. 1I). In contrast, NSP14 significantly increases m^7^G while both m^5^C and m^6^A levels are minimally altered, suggesting that NSP14 specifically induces internal m^7^G modification in mRNA. Together, these results demonstrate that NSP14 significantly increases m^7^G modification in the internal sequence of mRNA.

### SARS-CoV-2 infection increases cellular m^7^G signal

**Figure 2.**
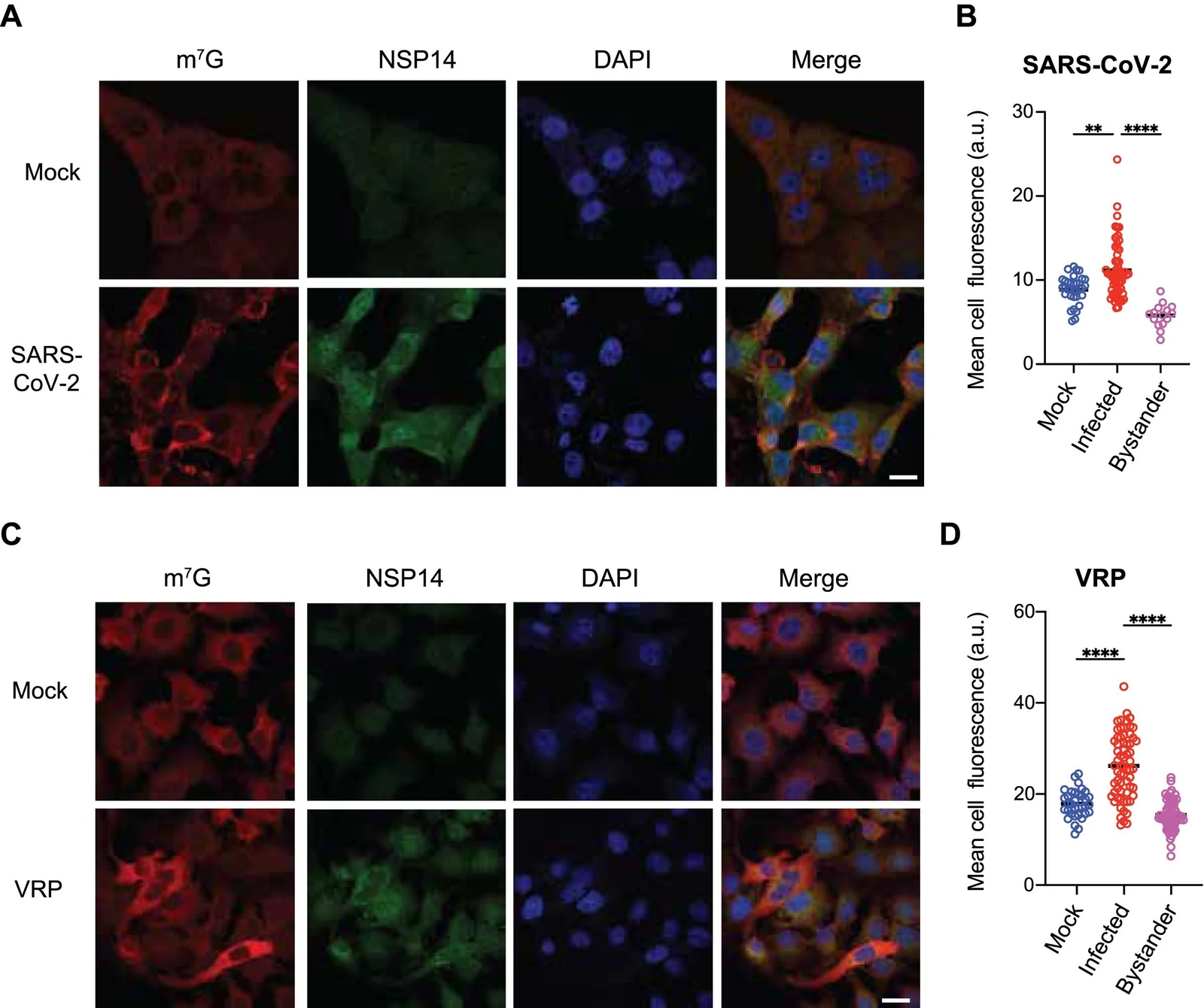
SARS-CoV-2 infection and viral replicon particles increase cellular m^7^G signal. (**A**) Immunofluorescence analysis of mock-infected and SARS-CoV-2-infected Vero E6 cells. Cells were infected with SARS-CoV-2 (MOI of 0.01 for 48 h), fixed, and stained with antibodies against m^7^G and SARS-CoV-2 NSP14. Representative images show increased m^7^G signal in NSP14-positive infected cells compared with mock-infected cells. Scale bar, 25 µm. (**B**) Quantification of m^7^G fluorescence intensity from individual cells is shown in (A). (**C**) Immunofluorescence analysis of Huh7 cells exposed to single-cycle SARS-CoV-2 VRPs for 24 h. Cells were fixed and stained with anti-m^7^G antibody, and VRP-positive cells were identified by NSP14 signal. Scale bar, 25 µm. (**D**) Quantification of m^7^G fluorescence intensity from individual cells is shown in (C). For (B) and (D), data are shown as single-cell fluorescence intensity. **P < 0.01, ****P < 0.001 by unpaired Student’s *t*-test.

To determine whether increased m^7^G signal also occurs in the context of viral infection, we performed immunofluorescence analysis in cells infected with SARS-CoV-2 or exposed to single-cycle SARS-CoV-2 VRPs. Since viral protein staining was used to identify virus-positive cells in these experiments, we first validated anti-NSP14 and anti-NSP10 antibodies by immunoblotting and confocal microscopy. The results show that the GeneTex antibody detected NSP14 protein by immunoblotting and exhibited strong co-localization with tagged NSP14 signals in confocal microscopy, supporting its use to identify NSP14-positive cells (Supplementary Fig. S2). For SARS-CoV-2 infection, Vero E6 cells were mock-infected or infected with SARS-CoV-2 (USA/WA-1/2020) and stained with antibodies against m^7^G and NSP14. Confocal microscopy analysis showed that SARS-CoV-2-infected cells exhibited increased m^7^G signal compared with mock-infected cells (Fig. 2A). This increase was consistently observed in cells that stained positive for viral NSP14 (Fig. 2B), suggesting an association between viral infection and an elevated cellular m^7^G signal.

To further validate this result in an independent viral replication system, we analyzed cells exposed to single-cycle SARS-CoV-2 VRPs [36] and stained with antibodies against m^7^G and NSP14. This VRP system delivers a propagation-defective SARS-CoV-2 replicon that supports viral RNA replication and viral protein expression in cells, but does not produce infectious progeny. Consistent with authentic viral infection, VRP-positive cells showed increased m^7^G signals compared with control cells (Fig. 2C, D). Together, these results show that SARS-CoV-2 infection and VRP replication are associated with increased cellular m^7^G signal, supporting the relevance of NSP14-induced m^7^G in the context of viral replication.

### *N*7-MTase activity is required to induce m^7^G modification

**Figure 3.**
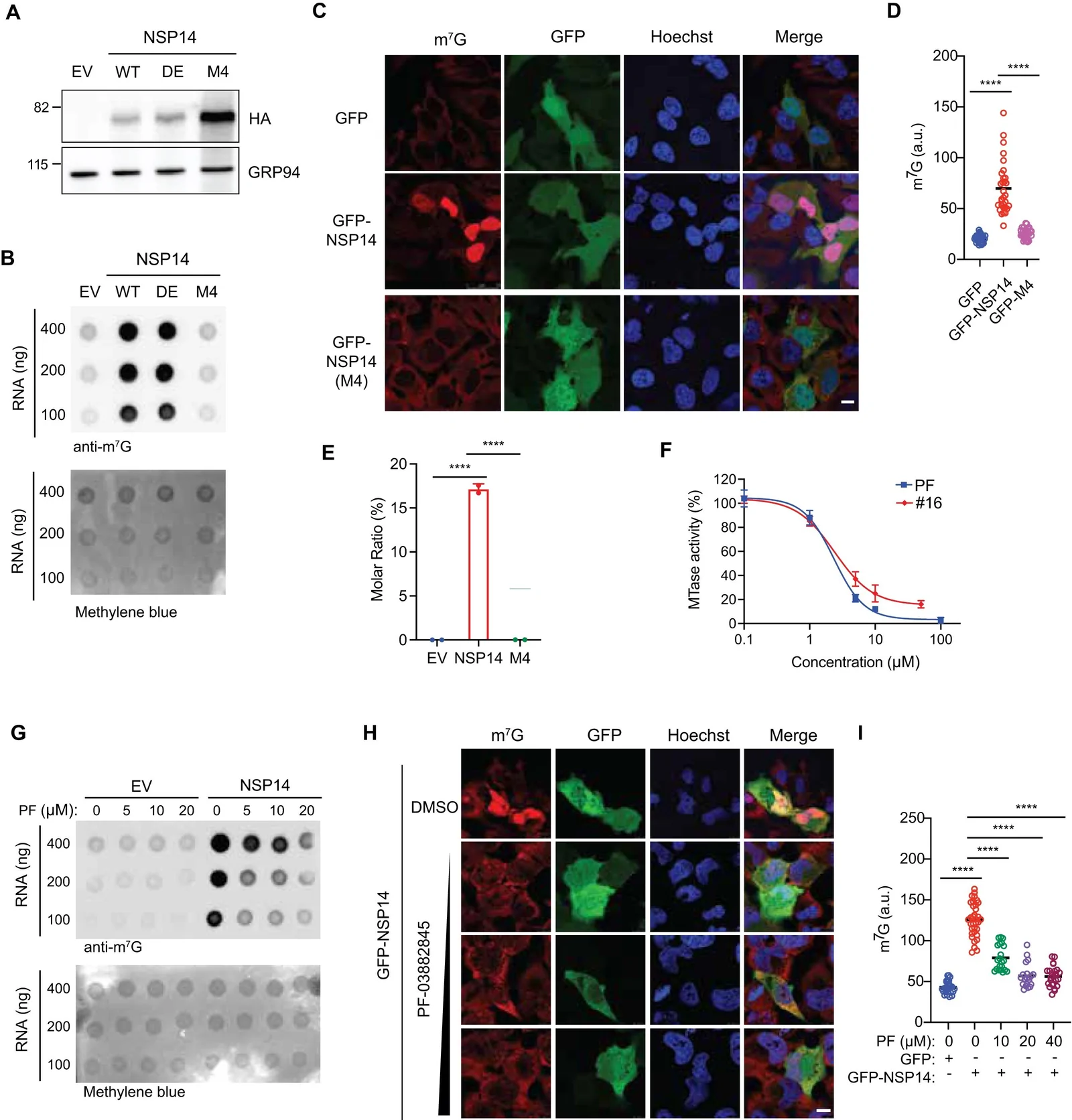
*N*7-MTase activity is required to induce m^7^G modification. (**A**) Immunoblot analysis of HEK293T cells transfected with EV, WT NSP14, the exonuclease mutant (D90A/E92A; DE), or the *N*7-methyltransferase mutant (M4). After 24 h of transfection, cell lysates were subjected to immunoblot analysis with anti-HA and anti-GRP94 antibodies. (**B**) RNA m^7^G immunoblot analysis of total RNA from transfected cells shown in (A). After 24 h of transfection, total RNA was extracted with TRIzol and subjected to m^7^G immunoblotting. Methylene blue staining was used as a loading control. (**C**) Confocal microscopy analysis of HeLa cells transfected with plasmids encoding GFP or GFP-tagged WT and NSP14 mutants. After 24 h of transfection, cells were fixed and stained with anti-m^7^G antibody (red) and Hoechst (blue). Scale bar, 10 µm. (**D**) Quantification of m^7^G fluorescence intensity from individual GFP-positive cells shown in (C). Data are shown as mean fluorescence intensity. ****P < 0.001 by unpaired Student’s *t*-test. a.u., arbitrary unit. (**E**) LC-MS/MS analysis of m^7^G modification in decapped, poly(A)-selected RNA from HEK293T cells transfected with EV, NSP14, or M4 for 24 h. RNA was extracted with TRIzol, isolated using oligo(dT), decapped with MDE, digested into nucleosides, and analyzed by LC-MS/MS. The results represent the molar ratio of modified to unmodified nucleosides. Data are shown as the mean ± SD of two biological replicates. ****P < 0.001 by unpaired Student’s *t*-test. (**F**) *In vitro* MTase activity assay using the recombinant SARS-CoV-2 NSP14–NSP10 complex and GTP as a substrate. The *N*7-MTase inhibitors, PF-03882845 (PF) and Compound 16 (#16), were used to inhibit *N*7-MTase activity. Data are shown as the mean ± SD of three biological replicates. (**G**) Dose-dependent inhibition of NSP14-induced m^7^G RNA modification in HEK293T cells treated with increasing concentrations of PF. Cells were transfected with EV or WT NSP14 and treated with PF for 24 h. Total RNA was extracted with TRIzol and subjected to m^7^G immunoblotting. (**H**) Confocal microscopy images of HeLa cells transfected with GFP–NSP14 and treated with PF (10, 20, and 40 µM). After 24 h, cells were fixed and stained with m^7^G antibody (red) and Hoechst (blue). Scale bar, 10 µm. (**I**) Quantification of m^7^G fluorescence intensity from individual GFP-positive cells shown in (H). Data are shown as mean fluorescence intensity. ****P < 0.001 by unpaired Student’s *t*-test.

NSP14 is a versatile enzyme with two distinct catalytic domains. The N-terminal 3′ to 5′ ExoN domain removes incorrectly incorporated nucleotides during viral RNA replication, which plays a critical role in maintaining high RNA replication fidelity [50]. The C-terminal domain exhibits *N*7-MTase activity, which modifies viral RNA at the 5′ cap guanosine, catalyzing the formation of the m^7^G cap structure (cap-0) [29]. Notably, mutations that inactivate either domain significantly impair viral replication [29, 50]. To determine the role of these activities in RNA m^7^G modification, we used two NSP14 mutants, D90A/E92A and D331A/G333A (M4), which disrupt the ExoN and *N*7-MTase activities, respectively [26, 51, 52]. We transfected HEK293T cells with either wild-type (WT) NSP14 or the mutants. Consistent with our previous study [26], M4 showed higher protein expression levels than the WT (Fig. 3A). To assess their roles in RNA modification, total RNA was extracted from the transfected cells and analyzed by m^7^G immunoblotting. The results showed that the *N7*-MTase mutant M4, but not the ExoN mutant, lost the m^7^G modification activity (Fig. 3B). Immunofluorescence analysis further demonstrated that M4 failed to induce m^7^G modification (Fig. 3C, D). To further study the role of *N*7-MTase in internal m^7^G modification of mRNA, RNA was extracted from HEK293T cells transfected with NSP14 and the M4 mutant, isolated using oligo(dT), decapped with MDE, and subjected to LC-MS/MS analysis. The results demonstrated that the *N*7-MTase mutant failed to induce internal m^7^G modification in mRNA (Fig. 3E).

To confirm the role of *N*7-MTase, we utilized two *N*7-MTase inhibitors of SARS-CoV-2 NSP14, PF-03882845 and Compound 16 (#16) [30, 53]. Using *in vitro* MTase assays, we demonstrated that PF-03882845 and #16 reduce NSP14 MTase activity, with IC_50_ values of 2.35 and 2.39 μM, respectively (Fig. 3F). To further investigate the role of *N*7-MTase activity in m^7^G modification, we treated HEK293T and HeLa cells with PF-03882845 and transfected the cells with NSP14. Using m^7^G immunoblotting, we showed that PF-03882845 treatment significantly reduced RNA m^7^G modification in NSP14-transfected cells in a dose-dependent manner (Fig. 3G). Similar results were observed in HeLa cells transfected with GFP–NSP14 using immunofluorescence staining (Fig. 3H, I). Consistent with the literature [30], we confirmed that PF-03882845 exhibited no detectable cytotoxicity under the experimental conditions (Supplementary Fig. S3). Together, these findings suggest that *N*7-MTase activity is required for internal m^7^G modification.

### The NSP10–NSP14 interaction increases internal m^7^G modification

**Figure 4.**
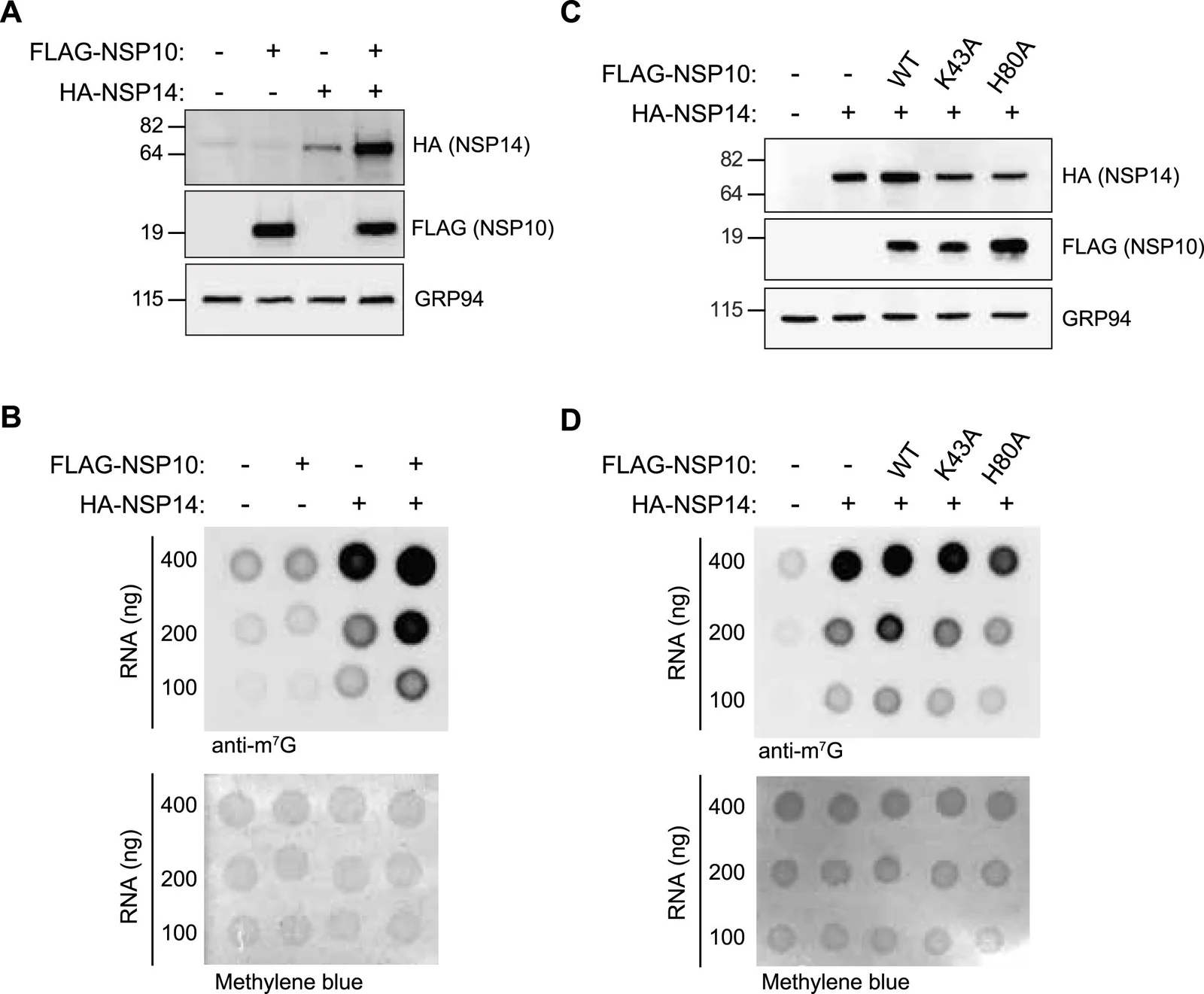
The NSP10–NSP14 interaction increases NSP14 abundance and internal m^7^G modification. (**A**) Immunoblot analysis of HEK293T cells co-transfected with plasmids encoding HA-tagged NSP14 (HA-NSP14) and FLAG-tagged NSP10 (FLAG-NSP10) for 24 h. Cell lysates were subjected to immunoblot analysis with anti-HA, anti-FLAG, and anti-GRP94 antibodies. (**B**) RNA m^7^G immunoblot analysis of HEK293T cells transfected with the indicated plasmids. After 24 h, total RNA was extracted with TRIzol and subjected to m^7^G immunoblotting and methylene blue staining. (**C**) Immunoblot analysis of HEK293T cells transfected with plasmids encoding HA-NSP14, FLAG-NSP10, or its mutants (K43A and H80A) for 24 h. Cell lysates were subjected to immunoblot analysis with anti-HA, anti-FLAG, and anti-GRP94 antibodies. (**D**) RNA m^7^G immunoblot analysis of HEK293T cells transfected with the indicated plasmids. After 24 h, total RNA was extracted with TRIzol and subjected to m^7^G immunoblotting and methylene blue staining.

SARS-CoV NSP14 protein forms a complex with the viral protein NSP10, which enhances NSP14 stability and enzymatic activity [24, 51]. Previous studies revealed the interaction in the SARS-CoV-2 homologs and that the formation of the complex significantly enhances the activities of NSP14 in ExoN and translational regulation [26, 54]. To study the role of NSP10 in NSP14-induced m^7^G modification, we co-transfected HEK293T cells with plasmids encoding NSP10 and NSP14. Protein levels of NSP10 and NSP14 were assessed by immunoblotting, which confirmed that co-expression with NSP10 significantly enhanced NSP14 expression (Fig. 4A), consistent with our previous findings [26]. Using m^7^G immunoblotting, we found that co-transfection of NSP10 and NSP14 increased m^7^G signal (Fig. 4B). We further confirmed the spatial association between these two viral proteins by confocal immunofluorescence microscopy. In cells co-transfected with HA-tagged NSP14 and FLAG-tagged NSP10, anti-NSP14 and anti-NSP10 staining showed overlapping perinuclear/cytoplasmic signals (Supplementary Fig. S4A). To determine the role of NSP10–NSP14 interaction in m^7^G modification, we used NSP10 mutants that have compromised protein–protein interaction. We previously showed that two NSP10 mutations at the protein–protein interface led to reduced (K43A) or no interaction (H80A) with NSP14 [26]. Consistently, compromising NSP10–NSP14 interaction resulted in reduced NSP14 expression (Fig. 4C). Notably, the impaired interaction led to reduced m^7^G modification (Fig. 4D).

To determine whether NSP10 increases m^7^G modification by directly enhancing NSP14 *N7*-MTase activity or indirectly by increasing NSP14 protein abundance, we performed a plasmid titration experiment to compare conditions with similar NSP14 expression levels. A fixed amount of NSP10 plasmid was co-transfected with increasing amounts of NSP14 plasmid. An empty vector was used to normalize total plasmid input. Immunoblot analysis showed that co-transfection of 125 ng of NSP14 with NSP10 produced NSP14 protein levels comparable with those observed with 500 ng of NSP14 alone (Supplementary Fig. S4B). We therefore compared these matched expression conditions by m^7^G immunoblotting. The results showed that 500 ng of NSP14 alone and 125 ng of NSP14 plus NSP10 induced similar levels of m^7^G modification (Supplementary Fig. S4C, D). Consistently, flow cytometry-based m^7^G (m^7^G FACS) analysis showed comparable m^7^G mean fluorescence intensity (Supplementary Fig. S4E). Together, these results suggest that the formation of the NSP10–NSP14 complex stabilizes NSP14 and increases its protein level and the RNA m^7^G modification.

### The internal m^7^G modification activity is conserved in coronaviruses

**Figure 5.**
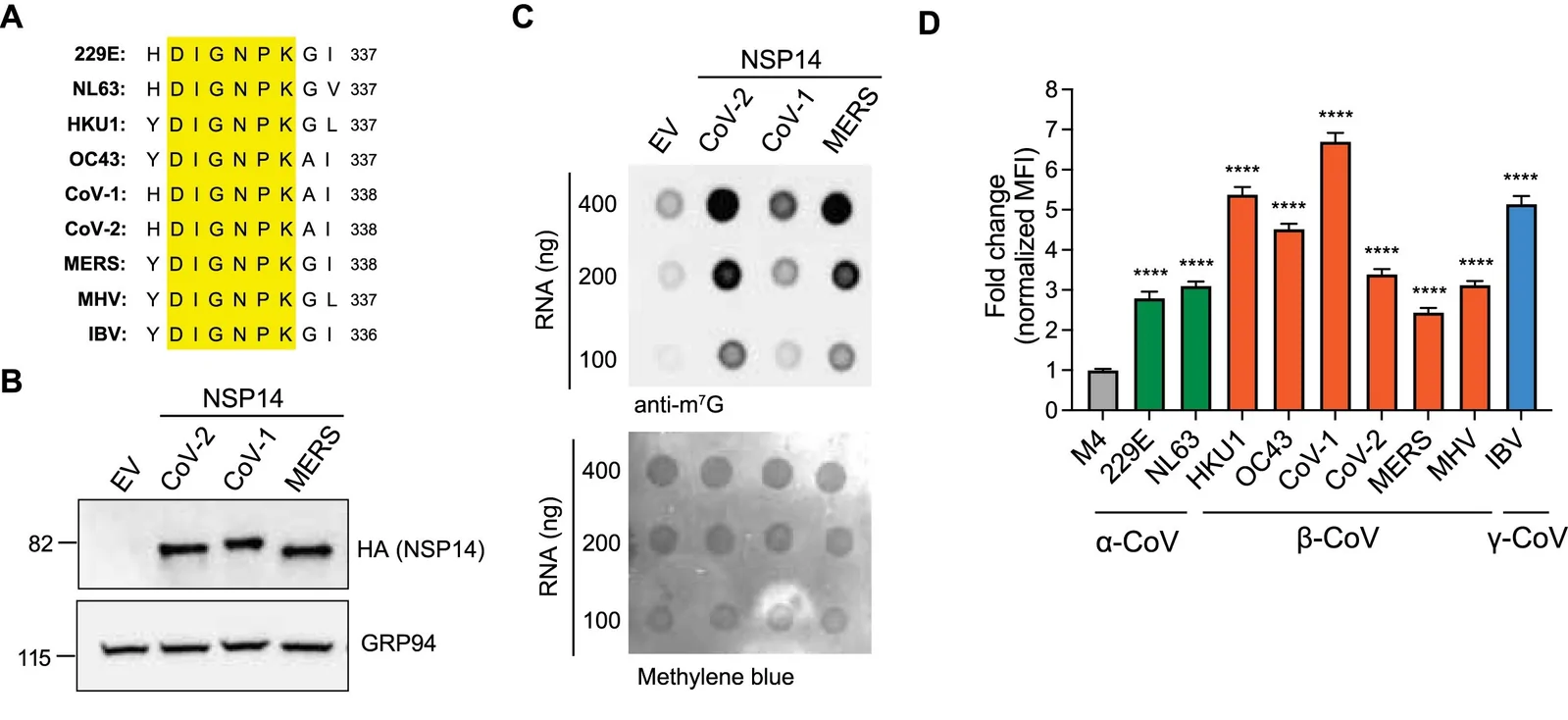
The internal m^7^G activity is conserved in coronaviruses. (**A**) Protein sequence alignment of coronavirus NSP14 at the *N*7-MTase active site. Conserved amino acid residues are highlighted in yellow. Human alphacoronavirus [HCoV-229E (229E), HCoV-NL63 (NL63)], human betacoronaviruses [HCoV-HKU1 (HKU1), HCoV-OC43 (OC43), MERS-CoV (MERS), SARS-CoV (CoV-1), SARS-CoV-2 (CoV-2)], murine betacoronavirus [mouse hepatitis virus (MHV)], and avian gammacoronavirus [infectious bronchitis virus (IBV)]. (**B**) Immunoblot analysis of HEK293T cells transfected with plasmids encoding different HA-tagged coronavirus NSP14 proteins for 24 h. Cell lysates were subjected to immunoblot analysis with anti-HA and anti-GRP94 antibodies. (**C**) RNA m^7^G immunoblot analysis of HEK293T cells transfected with the indicated plasmids. After 24 h, total RNA was extracted with TRIzol and subjected to m^7^G immunoblotting and methylene blue staining. (**D**) Flow cytometry analysis of HEK293T cells transfected with plasmids encoding HA-tagged NSP14 proteins from different coronaviruses for 24 h. Cells were fixed, stained with anti-m^7^G and anti-HA antibodies, and analyzed by flow cytometry. Data are presented as the mean ± SD of 3–9 biological replicates. ****P < 0.001 by unpaired Student’s *t*-test.

The *N*7-MTase activity of NSP14 is essential for the viral RNA 5′-end modification in human coronaviruses (HCoVs), which plays a critical role in viral replication and evasion of host immune responses [29, 55]. Disruption of *N*7-MTase activity, through either mutations or chemical inhibitors, significantly impairs viral replication [29, 30, 31]. Sequence analysis of CoV NSP14 proteins reveals high sequence homology (Supplementary Fig. S5). Notably, the SAM-binding motif (DxGxPxG/A) in the *N*7-MTase domain [29] is conserved within CoVs (Fig. 5A). To study whether this *N*7-MTase activity extends to RNA m^7^G modification, we transfected HEK293T cells with NSP14 from different CoVs. First, we examined highly pathogenic human betacoronaviruses, including SARS-CoV, SARS-CoV-2, and Middle East respiratory syndrome coronavirus (MERS-CoV). The protein expression levels were determined by immunoblotting (Fig. 5B). Using m^7^G immunoblotting, we found significant increases in RNA m^7^G modification induced by these NSP14 homologs (Fig. 5C), suggesting a conserved RNA m^7^G modification activity.

To further screen the RNA modification activity, we developed a flow cytometry approach using m^7^G antibody (Supplementary Fig. S6A). In brief, HEK293T cells were transfected with plasmids encoding HA-tagged NSP14. RNA m^7^G modification at the single-cell level was assessed by intracellular staining with m^7^G antibody. Flow cytometry analysis revealed that, at the single-cell level, SARS-CoV-2 NSP14 significantly increased the m^7^G signal compared with the *N*7-MTase mutant (Supplementary Fig. S6B). We applied this method to assess the m^7^G modification activity of NSP14 from coronaviruses. The results confirmed that NSP14 proteins from highly pathogenic human betacoronaviruses exhibited m^7^G modification activity (Fig. 5D). Moreover, we found similar activity in NSP14 from the common cold human alpha- and betacoronaviruses associated with mild to moderate, self-limiting respiratory diseases, including HCoV-229E, HCoV-NL63, HCoV-HKU1, and HCoV-OC43 (Fig. 5D). Interestingly, this activity is also conserved in a murine betacoronavirus, mouse hepatitis virus (MHV), and an avian gammacoronavirus, avian infectious bronchitis virus (IBV) (Fig. 5D). Together, these results suggest that the m^7^G modification is a conserved activity of NSP14 proteins across coronaviruses.

### NSP14 catalyzes the conversion of GTP into m^7^GTP

Internal m^7^G modifications are typically catalyzed by a group of *N*7-MTases, commonly known as m^7^G writers, which directly methylate RNA at specific sites [10]. However, NSP14 is a viral *N*7-MTase best characterized for methylating guanosine-containing cap-like substrates, and previous studies showed that SARS-CoV NSP14 can catalyze the conversion of GTP to m^7^GTP *in vitro* [56]. In addition, cellular expression of SARS-CoV-2 NSP14 is associated with increased intracellular m^7^GTP [27]. These results suggest that NSP14-induced internal m^7^G modification may occur through a nucleotide pool mechanism, in which NSP14 generates m^7^GTP as an aberrant nucleotide substrate for transcription. We therefore hypothesized that NSP14-derived m^7^GTP is incorporated into nascent RNA by cellular RNA polymerases, leading to internal m^7^G modification.

**Figure 6.**
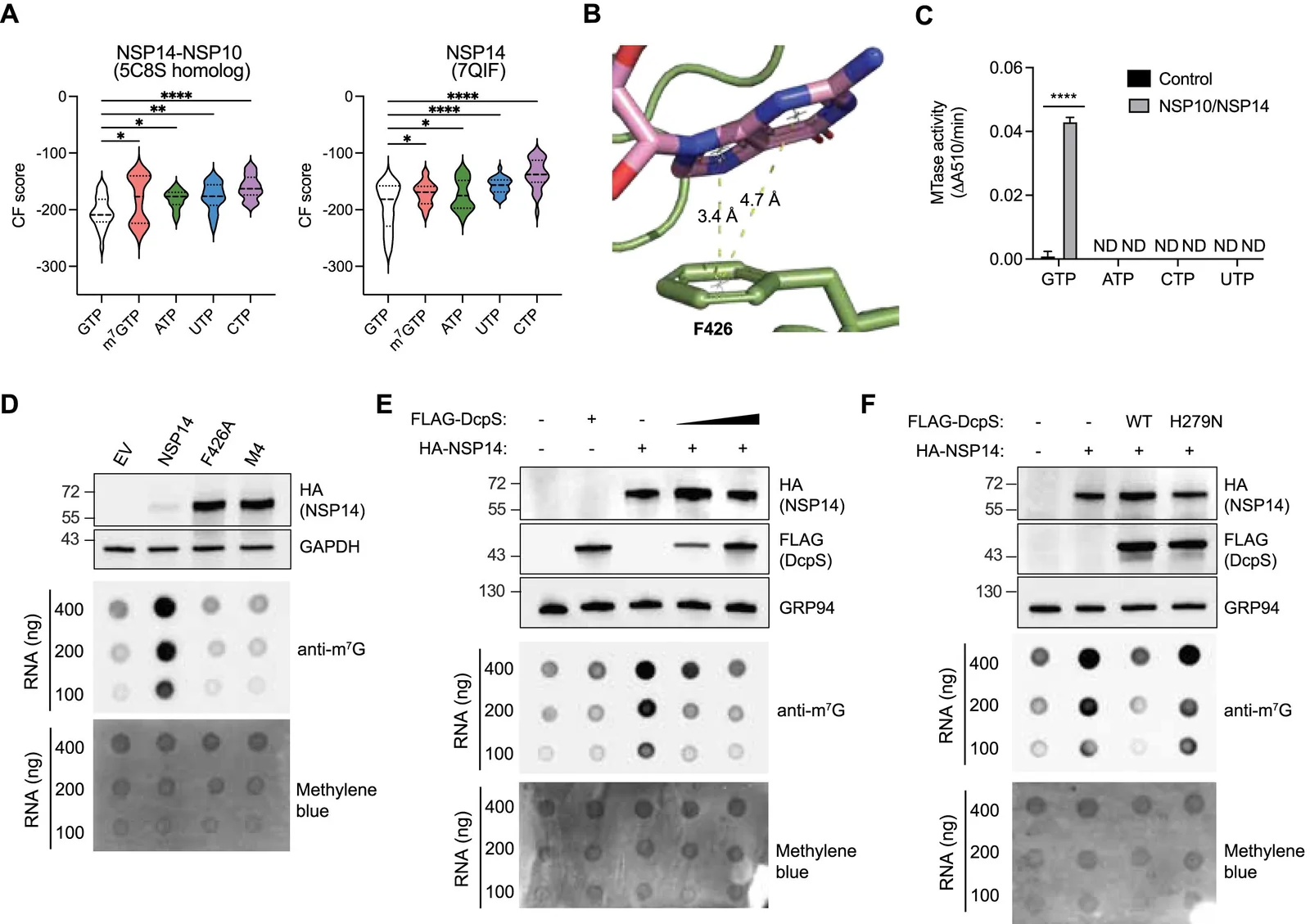
NSP14 methylates GTP to generate m^7^GTP for RNA modification. (**A**) Molecular docking analysis of SARS-CoV-2 NSP14 with various NTPs, illustrating differential binding affinities. The complementary function (CF) score is a docking metric that reflects the predicted binding affinity; more negative values indicate stronger binding. Data are shown as violin plots representing the top five poses from five independent docking runs (25 poses in total). *P < 0.05, **P < 0.01, ****P < 0.001 by unpaired Student’s *t*-test. (**B**) Structural model highlighting the π–π stacking interaction between the guanine base of GTP (pink) and residue F426 of NSP14 (green). (**C**) *In vitro* MTase activity assay of the NSP14–NSP10 complex using NTPs. Data are presented as the mean ± SD of three biological replicates. ****P < 0.001 by unpaired Student’s *t*-test. ND, not detected. (**D**) Protein and RNA m^7^G immunoblot analyses of HEK293T cells transfected with WT, F426A, and M4 mutant NSP14 for 24 h. Cell lysates were subjected to immunoblot analysis with anti-HA and anti-GAPDH antibodies. Total RNA was extracted with TRIzol and subjected to m^7^G immunoblotting and methylene blue staining. (**E**) Protein and RNA m^7^G immunoblot analyses of HEK293T cells co-transfected with plasmids encoding HA-tagged NSP14 (HA-NSP14) and FLAG-tagged DcpS (FLAG-DcpS) for 24 h. (**F**) Protein and RNA m^7^G immunoblot analyses of HEK293T cells co-transfected with plasmids encoding HA-NSP14 and WT or inactive DcpS mutant (H279N) for 24 h.

To study the structural basis of this activity, we first employed homology modeling and molecular docking to study the binding interactions of SARS-CoV-2 NSP14 with nucleotide triphosphates (NTPs), GTP, ATP, CTP, and UTP. We used two structural templates, SARS-CoV-2 NSP14 bound to the cap analog m^7^GpppG (PDB 7QIF) and the SARS-CoV-1 NSP14–NSP10 complex with GpppA (PDB 5C8S). To model SARS-CoV-2-specific interactions in the SARS-CoV structure, we substituted homologous amino acids. Per-residue interaction energies were calculated to assess the contribution of individual residues, and docking simulations were repeated five times. Analysis of available NSP14 structures showed that the nucleotide-binding cavity is structurally conserved across SARS-CoV and SARS-CoV-2 NSP14 complexes, supporting the use of this binding site geometry for docking (Supplementary Fig. S7). The molecular docking results showed that, in both structures of NSP14 and the NSP14–NSP10 complex, the complementary function (CF) score of GTP had a larger negative value than other NTPs, suggesting that GTP has the strongest predicted binding affinity among the tested NTPs, followed by ATP, UTP, and CTP (Fig. 6A). In contrast, pyrimidine-based NTPs such as UTP and CTP lacked this interaction and fell outside the binding cavity (Supplementary Fig. S8A). Moreover, the *N*7-methylated guanine of m^7^GTP was positioned outside the binding cavity (Supplementary Fig. S8B), and docking analyses predicted that m^7^GTP bound less favorably than GTP (Fig. 6A), consistent with its function as a product released from the NSP14 active site. Because SAM is the methyl donor required for NSP14-mediated methylation and occupies the catalytic site, we further evaluated whether SAM would alter the predicted NTP-binding mode. To do this, we repeated the docking with SAM included as a fixed molecule in the active site. The presence of SAM produced similar relative NTP scoring (Supplementary Fig. S8C), suggesting that the predicted preference for GTP is maintained when SAM is included in the model. The docking poses further suggested that the guanine base of GTP is positioned near the aromatic side chain of F426 at distances consistent with a potential π–π stacking interaction (Fig. 6B; Supplementary Fig. S8D).

To validate the modeling results, we examined the catalytic activity of NSP14 in synthesizing m^7^GTP. We conducted an *in vitro* methylation assay using NTPs. The results demonstrated that NSP14 methylated GTP but not ATP, UTP, and CTP (Fig. 6C). The identity of the methylated product was confirmed as m^7^GTP by mass spectrometric analysis (Supplementary Fig. S9). To test the role of F426 in RNA m^7^G modification, we transfected HEK293T cells with plasmids encoding WT and F426A NSP14, and assessed m^7^G using immunoblotting. The results demonstrated that F426A significantly reduced NSP14-induced m^7^G (Fig. 6D), suggesting a critical role for F426 in substrate recognition and catalysis. Notably, F426 is conserved among coronaviruses, supporting its importance for viral replication.

To further confirm the involvement of m^7^GTP in m^7^G modification in cells, we employed the scavenger mRNA decapping enzyme DcpS, a scavenger decapping enzyme that primarily hydrolyzes residual cap structures or capped oligonucleotides generated during RNA decay and can convert cellular m^7^GTP to m^7^GMP [27]. We co-transfected HEK293T cells with plasmids encoding NSP14 and DcpS, and assessed m^7^G using immunoblotting. The results demonstrated that DcpS significantly reduced NSP14-induced m^7^G in a dose-dependent manner (Fig. 6E). Moreover, we also generated an inactive mutant of DcpS, H279N [57]. The results showed that H279N failed to inhibit NSP14-induced m^7^G (Fig. 6F), suggesting that m^7^GTP is required for the NSP14-induced m^7^G. Together, these molecular modeling and experimental results demonstrate that NSP14 catalyzes the synthesis of m^7^GTP from GTP. This product is then required, probably through incorporation by RNA Pols, for internal RNA m^7^G modification.

### NSP14 induces internal m^7^G via RNA polymerase-dependent incorporation

**Figure 7.**
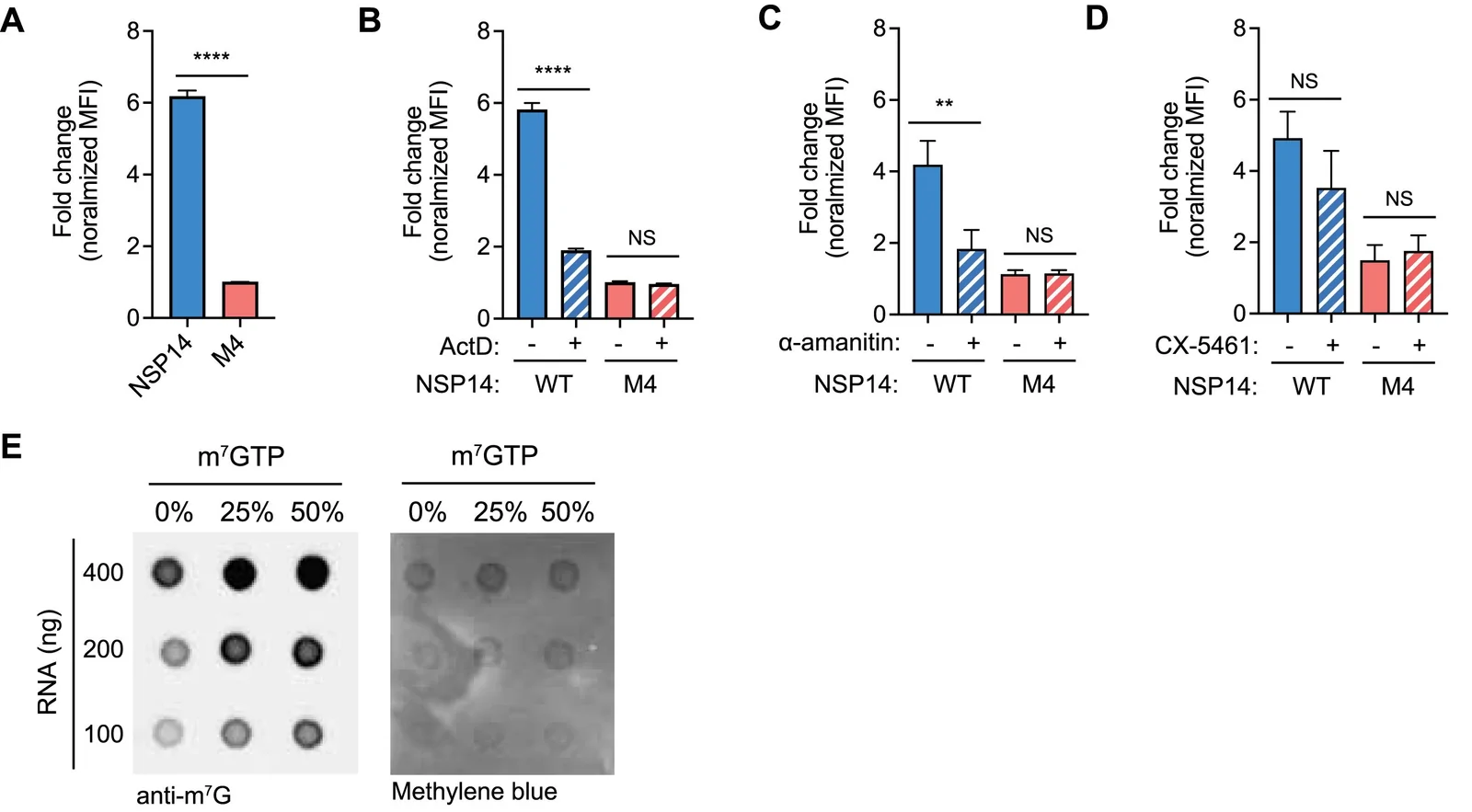
RNA polymerase II incorporates m^7^GTP into RNA. (**A**) Flow cytometry analysis of HEK293T cells transfected with *in vitro* transcribed RNA encoding NSP14 or M4 for 24 h. Cells were fixed, stained with anti-m^7^G and anti-HA antibodies, and analyzed by flow cytometry. Data are presented as the mean ± SD of three biological replicates. ****P < 0.001 by unpaired Student’s *t*-test. (**B**–**D**) Flow cytometry analysis of HEK293T cells transfected with *in vitro* transcribed RNA encoding NSP14 or M4 and treated with (B) actinomycin D (ActD), (C) α-amanitin, or (D) CX-5461 for 24 h. Cells were fixed, stained with anti-m^7^G and anti-HA antibodies, and analyzed by flow cytometry. Data are presented as the mean ± SD of three biological replicates. **P < 0.01, ****P < 0.001 by unpaired Student’s *t*-test. NS, not significant. (**E**) RNA m^7^G immunoblot analysis of *in vitro* transcribed RNA. RNA was synthesized using HeLa nuclear extract from a CMV promoter-driven DNA fragment, with m^7^GTP supplemented at the indicated ratios. The resulting RNA was analyzed by m^7^G immunoblotting and methylene blue staining. The background m^7^G signals observed at 0% m^7^GTP are due to endogenous RNA in the HeLa nuclear extract.

To study the role of RNA Pol activity in NSP14-induced m^7^G, we used transcription inhibitors to block RNA synthesis in cells. Since these inhibitors can suppress transcription from plasmid DNA, we instead transfected HEK293T cells with *in vitro* transcribed mRNA encoding either WT NSP14 or the *N*7-MTase inactive M4 mutant. This design bypasses cellular transcription of the NSP14 constructs and minimizes the possibility that transcription inhibitors reduce NSP14 expression by inhibiting plasmid-derived NSP14 transcription. Consistent with results from plasmid transfection, NSP14 mRNA transfection induced m^7^G modification, whereas the M4 mutant failed to exhibit this activity (Fig. 7A). To inhibit transcription, HEK293T cells were treated with actinomycin D, a broad-spectrum transcription inhibitor, followed by transfection with NSP14 mRNA. The results showed that actinomycin D significantly reduced m^7^G modification (Fig. 7B), indicating an important role for active transcription in NSP14-induced m^7^G. Given that NSP14 increases m^7^G levels in mRNA (Fig. 1G, I), we next examined the effects of selectively inhibiting RNA Pol II activity using α-amanitin. The results demonstrated that α-amanitin treatment significantly inhibited m^7^G modification (Fig. 7C). In contrast, treatment with CX-5461, an RNA Pol I inhibitor, had no effect on m^7^G modification (Fig. 7D). Additionally, we confirmed that the transcription inhibitors did not significantly affect NSP14 or M4 expression or cause cytotoxicity under these conditions (Supplementary Fig. S10A, B). The effectiveness of the inhibitors was confirmed by RT–qPCR analysis of genes transcribed by each RNA Pol: 47S rRNA (Pol I), Myc (Pol II), and U6 snRNA (Pol III) (Supplementary Fig. S10C, D).

To study the role of RNA Pol II in m^7^GTP incorporation into mRNA, we used a human *in vitro* transcription system with HeLa nuclear extract. In brief, we employed a DNA template containing a cytomegalovirus (CMV) promoter, which drives expression of the enhanced GFP (EGFP) gene in a Pol II-dependent manner. To assess the incorporation of m^7^GTP, we supplemented the *in vitro* transcription reaction with m^7^GTP and subsequently analyzed the *in vitro* transcribed RNA using m^7^G immunoblotting. The results showed that m^7^GTP was incorporated into RNA by RNA Pol II (Fig. 7E). Together, these results indicate that NSP14 induces internal m^7^G modification through a mechanism that depends on RNA Pol II-mediated incorporation of NSP14-generated m^7^GTP into mRNA.

### RNA m^7^G modification induces alternative splicing

**Figure 8.**
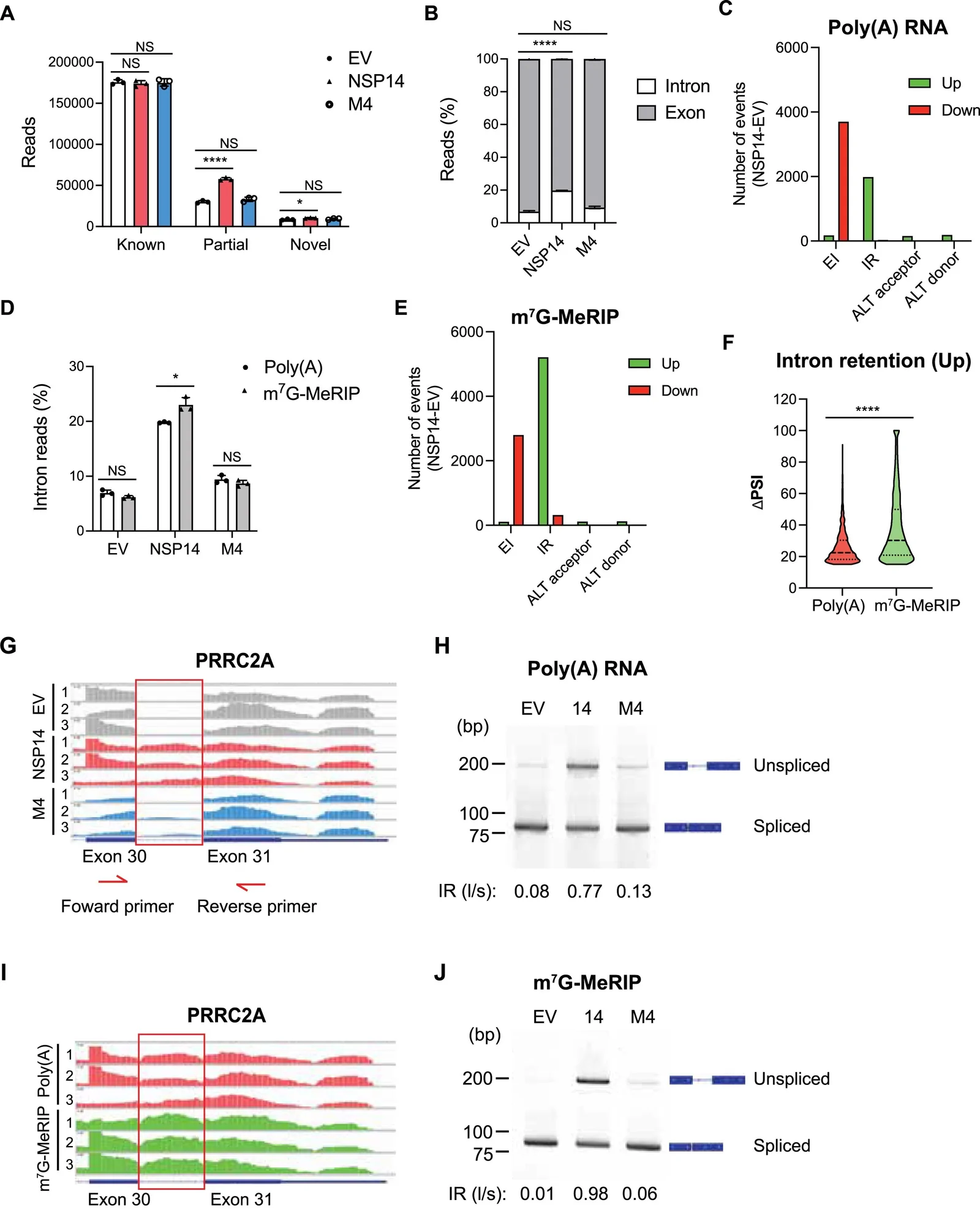
m^7^G modification induces alternative splicing. (**A**) Splice junction analysis of poly(A) RNA from HEK293T cells transfected with empty vector (EV), WT NSP14, or the *N*7-MTase mutant (M4) for 24 h. Poly(A) RNA was isolated using oligo(dT) from total RNA and subjected to RNA-seq analysis. NSP14 expression increased the number of partial novel and novel splicing junctions but not known junctions. (**B**) RSeQC read distribution analysis of poly(A) RNA from HEK293T cells transfected with EV, NSP14, or M4 for 24 h. Poly(A) RNA was isolated as described in (A). (**C**) Quantification of AS events in poly(A) RNA between NSP14- and EV-transfected cells. EI, exon inclusion; IR, intron retention; ALT, alternative. (**D**) RSeQC read distribution analysis of m^7^G-enriched mRNA from HEK293T cells expressing EV, NSP14, or M4 for 24 h. Poly(A) RNA was isolated using oligo(dT) from total RNA, then decapped with MDE, and immunoprecipitated by anti-m^7^G antibody to isolate m^7^G-MeRIP RNA. Both poly(A) RNA and m^7^G-MeRIP RNA were subjected to RNA-seq. (**E**) Quantification of AS events in m^7^G-enriched mRNA between NSP14- and EV-transfected cells. (**F**) Analysis of the level of NSP14-induced intron retention. Violin plots show the distribution of ΔPSI (percent spliced-in index) values for the up-regulated intron retention events in poly(A) RNA versus m^7^G-MeRIP RNA. ΔPSI values reflect the change in splicing inclusion levels between NSP14- and EV-transfected cells. NSP14 expression led to significantly higher ΔPSI values in m^7^G-modified RNA compared with poly(A) RNA. (**G**) RNA-seq tracks of PRRC2A showing increased intron reads (highlighted in a red rectangle) between exons 30 and 31 in cells expressing NSP14. Poly(A) RNA from HEK293T cells expressing EV, NSP14, or M4 were isolated as described in (A). Forward and reverse primers used for RT-PCR to detect intron retention are indicated. (**H**) RT-PCR analysis of intron retention in poly(A) RNA from HEK293T cells transfected with EV, NSP14, or M4 for 24 h. Poly(A) RNA was reverse-transcribed and amplified by PCR. The resulting PCR products were analyzed by DNA gel electrophoresis. The intron retention ratio of unspliced “long” fragments to spliced “short” fragments [IR(l/s)] was quantified using ImageJ. (**I**) RNA-seq tracks of PRRC2A comparing poly(A) RNA and m^7^G-MeRIP RNA, showing higher intron retention in m^7^G-enriched RNA. RNA was extracted and subjected to m^7^G-MeRIP-seq as shown in (D). (**J**) RT-PCR analysis of intron retention in m^7^G-MeRIP RNA from HEK293T cells transfected with EV, NSP14, or M4, as described in (H). m^7^G-MeRIP RNA, rather than poly(A) RNA, was used for RT-PCR. For (A), (B), (D), and (F), data are presented as the mean ± SD of three biological replicates. *P < 0.05, ****P < 0.001 by unpaired Student’s *t*-test. NS, not significant.

Betacoronaviruses, including SARS-CoV-2, alter host RNA splicing in both *in vitro* infection models and clinical COVID-19 samples [58]. Infected cells display an increased prevalence of intron-retaining transcripts [58]. In SARS-CoV-2 infection, these splicing changes affect a broad spectrum of genes and pathways, many of which are closely linked to RNA metabolism, viral replication, and host antiviral responses [58]. Expression of NSP14 mirrors a substantial fraction of the transcriptional changes observed during SARS-CoV-2 infection [27], suggesting that NSP14 is a key driver of host RNA remodeling. Moreover, NSP14 robustly promotes AS, particularly intron retention [27, 28]. Notably, the *N*7-MTase activity of NSP14 is required for this effect [27, 28]. To study the role of NSP14-induced m^7^G modification in AS, we first examined how NSP14 affects the formation of partial novel and novel splice junctions [59]. Partial novel splice junctions are splicing events in which one splice site (either the 5′ donor or 3′ acceptor) matches a known exon boundary, but the other site is unannotated. These events probably reflect mispairing between canonical and cryptic splice sites. Novel splice junctions, in which both splice sites are unannotated, represent entirely aberrant splicing events and indicate profound disruption of normal splicing regulation. RNA-seq analysis showed that expression of NSP14 induced a significant increase in partial novel and novel splice junctions, without affecting canonical splicing events (Fig. 8A). In contrast, cells expressing an *N*7-MTase-deficient mutant of NSP14 (M4) showed no such changes, suggesting that the *N*7-MTase activity of NSP14 is essential for altering host splicing patterns.

Among the major types of AS, previous studies demonstrated that NSP14 increases intron retention and reduces exon inclusion [27, 28], both of which contribute to an increased level of introns. Consistently, we showed that NSP14, but not the M4 mutant, significantly increased intron reads in poly(A) RNA (Fig. 8B), accompanied by elevated intron retention and reduced exon inclusion (Fig. 8C). To further study the role of m^7^G in AS, we performed m^7^G-methylated RNA immunoprecipitation (m^7^G-MeRIP) to enrich internal m^7^G-modified mRNA. In brief, mRNA was isolated using oligo(dT), decapped with MDE, and subjected to RNA immunoprecipitation using m^7^G antibody. RNA-seq analysis revealed that NSP14 induced an increase in intron reads in internal m^7^G-modified RNA (Fig. 8D). Notably, the intron levels were significantly higher in internal m^7^G-modified RNA compared with poly(A) RNA, suggesting an important role for m^7^G in modulating splicing patterns. Moreover, NSP14 induced more intron retention events in internal m^7^G-modified RNA compared with poly(A) RNA (Fig. 8C versus E). In addition, NSP14-induced m^7^G modifications not only increased intron retention events but also elevated the levels of retention (Fig. 8F).

Intron retention can generate aberrant protein isoforms and, in many cases, abolish protein function. To assess the biological significance of NSP14-induced intron retention, we performed GO analysis on genes with increased intron retention (Supplementary Fig. S11). Genes affected by NSP14 were strongly enriched for processes related to genome stability and RNA metabolism, including DNA replication and repair, cell cycle regulation, and RNA processing. Notably, many of these GO terms are also enriched among differentially spliced genes in SARS-CoV-2 infection [58], indicating that NSP14 expression recapitulates a substantial fraction of the alternative splicing changes observed during infection. At the cellular level, these genes localize predominantly to nuclear compartments associated with chromatin organization, splicing, and protein quality control. Functionally, they are enriched for enzymatic and regulatory activities acting on DNA and RNA, including helicases, transcription factors, and chromatin-modifying enzymes. Together, these results indicate that NSP14-induced intron retention broadly disrupts nuclear programs that coordinate genome maintenance with RNA processing, suggesting a mechanism by which the virus reshapes host gene expression to favor infection.

To validate the changes in AS, we performed reverse transcription followed by PCR to quantify intron retention levels in selected target genes. We focused on three genes, PRRC2A, ATG101, and RPS27A, which exhibited increased intron retention upon NSP14 expression, whereas SSBP3 served as a negative control (Supplementary Fig. S12A). For each gene, we designed a set of primers targeting a specific intron that displayed increased retention (Fig. 8G; Supplementary Fig. S12B). These primers span adjacent exon boundaries, producing ∼200 bp amplicons from unspliced transcripts, whereas shorter products are amplified from fully spliced mRNA (Fig. 8H; Supplementary Fig. S13). Using poly(A) RNA as templates, we found that NSP14 increased the abundance of long fragments compared with EV and M4, indicating an increase in unspliced RNA levels (Fig. 8H; Supplementary Fig. S13). Given that the m^7^G-MeRIP-seq results revealed a higher level of intron retention (Fig. 8E, F), we further analyzed the unspliced RNA levels using m^7^G-MeRIP RNA (Fig. 8I; Supplementary Fig. S14). For example, m^7^G-enriched RNA showed an increase in the intron reads between exons 30 and 31 of PRRC2A compared with poly(A) RNA (Fig. 8I). Consistently, RT-PCR results showed an increase in unspliced RNA levels, as indicated by an increase in the IR(l/s) ratio from 0.77 to 0.98 (Fig. 8H versus J). Together, these findings demonstrate that NSP14 induces intron retention in m^7^G-modified RNA.

To further validate the role of NSP14-induced m^7^G modification in AS, we utilized DcpS to reduce internal m^7^G modification levels. Cells were co-transfected with the plasmids encoding NSP14 and DcpS. The AS patterns of target genes were analyzed using RT-PCR. Co-transfection with DcpS reduced NSP14-induced intron retention, restoring splicing patterns to those observed in control conditions (Supplementary Fig. S15). Together, these findings demonstrate that NSP14 induces AS in m^7^G-modified RNA.

### Inhibiting NSP14-induced alternative splicing reduces SARS-CoV-2 replication

**Figure 9.**
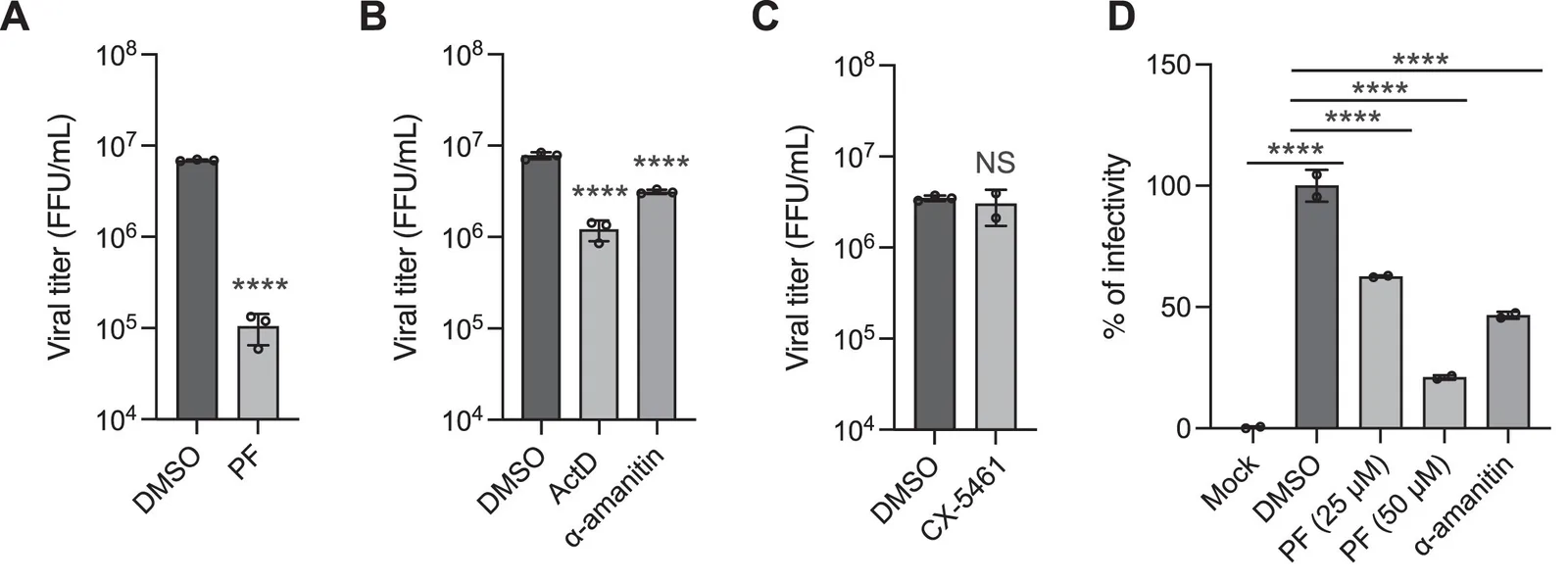
*N*7-MTase and transcription inhibitors reduce SARS-CoV-2 replication. (**A**–**C**) Vero E6 cells were infected with SARS-CoV-2 (WA1/2020) at an MOI of 1 and treated with the indicated inhibitors for 24 h. Cells were treated with (A) the NSP14 *N*7-MTase inhibitor PF-03882845 (PF) and (B and C) transcription inhibitors [actinomycin D (ActD), α-amanitin, and CX-5461]. Supernatants were collected, and viral titers were determined using focus-forming assays. FFU, focus-forming unit. (**D**) A549-Ace2-Dpp4-Tmprss2 cells were infected with the SARS-CoV-2 XEC variant at an MOI of 1 and treated with the indicated inhibitors for 24 h. Supernatants were collected, and viral titers were determined using an immunofluorescence-based viral replication assay. Data are presented as the mean ± SD of 2–3 biological replicates. ****P < 0.001 by unpaired Student’s *t*-test. NS, not significant.

A previous study identified a direct correlation between SARS-CoV-2 viral load and splicing isoform distribution in patient samples [58]. Higher viral loads are associated with significant shifts in AS patterns in SARS-CoV-2 infection, suggesting that inducing AS enhances viral replication. To study the role of NSP14-induced AS in SARS-CoV-2 replication, Vero E6 cells were infected with SARS-CoV-2 at an MOI of 1 for 24 h with *N*7-MTase or RNA Pol inhibitors. The cell culture medium was harvested to determine viral titers using focus-forming assays (FFAs). The results demonstrated that the *N*7-MTase inhibitor PF-03882845 significantly reduced SARS-CoV-2 replication (Fig. 9A), consistent with previous studies [30]. This result suggests the important role of the *N*7-MTase activity of NSP14 in viral replication.

As inhibition of the *N*7-MTase activity of NSP14 affects both viral RNA 5′ capping and host RNA m^7^G modification, we next focused on the role of NSP14-induced host m^7^G modification in viral replication. Since internal m^7^G modification is incorporated by RNA Pols (Fig. 7), we employed transcription inhibitors to selectively disrupt host m^7^G modification. The results showed that actinomycin D treatment significantly inhibited viral replication (Fig. 9B). Notably, inhibition of RNA Pol II with α-amanitin also led to a significant reduction in viral replication (Fig. 9B). In contrast, inhibition of RNA Pol I with CX-5461 did not affect viral replication (Fig. 9C). We confirmed that PF-03882845 and transcription inhibitors exhibited no detectable cytotoxicity under the experimental conditions (Supplementary Fig. S16).

To further assess the dependence of SARS-CoV-2 variants on RNA Pol II activity, we examined a clinical isolate of the XEC variant, an Omicron subvariant that accounted for an estimated 45% of US cases in early December 2024. Consistently, inhibition of RNA Pol II with α-amanitin significantly inhibits XEC replication (Fig. 9D). Together, these results are consistent with a model in which NSP14-induced AS and internal m^7^G modification are critical for efficient SARS-CoV-2 replication, and that disruption of these processes can substantially impair viral replication.

## Discussion

Coronavirus NSP14 plays canonical roles in viral RNA capping and proofreading via its *N*7-MTase and ExoN domains, respectively. Both enzymatic activities are indispensable for efficient viral replication, suggesting the essential role of NSP14 in the coronavirus life cycle [29, 50]. Beyond its functions on viral RNA, recent studies have revealed a versatile role for NSP14 in regulating host RNA metabolism. SARS-CoV-2 NSP14 alters host RNA splicing, inhibits RNA nuclear export, and suppresses host protein synthesis [26, 27, 28]. Here, we uncovered a novel function of NSP14 in reshaping host RNA epitranscriptomics and splicing patterns through internal m^7^G modification. The m^7^G modification activity is specific to the internal sequences of polyadenylated mRNA. Notably, this activity is conserved across multiple coronaviruses, including SARS-CoV-1, SARS-CoV-2, MERS-CoV, and other human and animal coronaviruses. Using an *N*7-MTase mutant and NSP14 inhibitors, we demonstrated that *N*7-MTase activity is essential for m^7^G modification. Mechanistically, NSP14 catalyzes the conversion of GTP into m^7^GTP, which is then incorporated into host mRNA by RNA Pol II. This reveals a novel mechanism by which viruses exploit the host transcriptional machinery to induce non-canonical RNA modifications. Importantly, this study also links NSP14-induced m^7^G modification to AS regulation. Inhibiting either the *N*7-MTase activity or m^7^G incorporation significantly impaired SARS-CoV-2 replication, suggesting that SARS-CoV-2 hijacks the host epitranscriptomic landscape to reprogram gene expression to favor viral replication. Together, this study provides new insight into the importance of RNA modifications in virus–host interactions and identifies NSP14 as a key driver of coronavirus-mediated epitranscriptomic changes.

SARS-CoV-2 NSP14 modulates host RNA metabolism by suppressing translation, blocking nuclear export, and remodeling the transcriptome [26, 27, 28]. These transcriptomic changes affect thousands of genes and include altered mRNA abundance and localization, disrupted splicing patterns, and up-regulation of circular RNAs, many of which are implicated in innate immunity. Notably, the *N*7-MTase activity of NSP14 is essential for these changes in RNA metabolism [26, 27, 28]. This activity, typically responsible for viral RNA capping, appears to have an unanticipated regulatory role in host gene expression. Moreover, through its *N*7-MTase activity, NSP14 activates inosine 5′-monophosphate dehydrogenase 2 (IMPDH2), the rate-limiting enzyme in *de novo* guanine nucleotide biosynthesis, leading to elevated GTP levels that drive metabolic and transcriptional changes, including NF-κB pathway activation and *CXCL8* induction [28]. Additionally, NSP14 disrupts mRNA splicing and nuclear export by targeting the host nuclear cap-binding complex (CBC), interfering with 5′ cap recognition [27]. This impairs the processing of pre-mRNAs and blocks their export to the cytoplasm. In this study, we demonstrated that NSP14 induces m^7^G modification in mRNA, which installs a methyl group at the *N*^7^ position of the guanine in the internal sequences. This modification can potentially alter RNA structure and RNA–protein interactions, contributing to changes in splicing, nuclear export, and translational regulation. Together, these findings reveal the *N*7-MTase domain of NSP14 as a multifunctional effector that orchestrates transcriptional reprogramming, impairs mRNA maturation, and supports viral immune evasion by disrupting host RNA metabolism at multiple levels.

Internal RNA modifications play critical roles in cellular function and human diseases. These modifications have been implicated in tumorigenesis, immune evasion, and reduced drug sensitivity, highlighting their potential as biomarkers and therapeutic targets in cancer and other diseases [60–63]. In viral infections, m^6^A modifications regulate viral replication and host immune responses [64]. Flavivirus infection induces m^6^A modifications in a subset of host mRNAs, regulating their expression through AS and translational regulation, resulting in a cellular environment that supports viral replication [65]. SARS-CoV-2 infection induces dynamic changes in the m^6^A methylome, with increased m^6^A methylation detected in both viral and host mRNA [66, 67]. For m^7^G modifications, stress conditions have been shown to induce internal mRNA modification that influences RNA translation and stability [68, 69]. Furthermore, a higher expression of most m^7^G-related genes in COVID-19 compared with non-COVID-19 patients has been found, suggesting a role in viral pathogenesis [70]. Here, we reported a novel function of SARS-CoV-2 NSP14 in internal m^7^G modification and AS. Importantly, inhibition of NSP14’s *N*7-MTase activity or blocking m^7^G incorporation impairs SARS-CoV-2 replication, reinforcing the idea that the virus manipulates the host epitranscriptome to establish a cellular environment conducive to viral replication. By reshaping the host mRNA landscape at the level of RNA modifications and splicing, NSP14 emerges not only as a key factor in viral replication but also as a modulator of host gene expression. Together, our findings expand the known repertoire of coronavirus–host interactions and identify the NSP14-induced internal m^7^G pathway as a critical axis of epitranscriptomic reprogramming with implications for viral pathogenesis, immune evasion, and potential therapeutic targeting.

RNA modifications are typically installed post-transcriptionally by specific enzymes known as “writers” [71]. These proteins recognize consensus RNA sequences or structural motifs and catalyze the corresponding chemical reactions [71]. Several cellular m^7^G writers are known to catalyze internal m^7^G modifications [72]. 18S rRNA is m^7^G modified at G1639 by the WBSCR22–TRM112 complex, whereas a subset of tRNAs is m^7^G modified at G46 by the METTL1–WDR4 complex [13–16]. These modifications play critical roles in RNA structure and translation initiation and fidelity [13–16]. mRNA also undergoes internal m^7^G modification by the METTL1–WDR complex, which plays important roles in translational regulation and stress responses [17–20]. Coronavirus NSP14 represents the first known viral protein that can induce internal m^7^G modification. Rather than acting as a conventional m^7^G writer, NSP14 methylates GTP into m^7^GTP, which is subsequently incorporated into mRNA by RNA Pol II. Additionally, through activation of IMPDH2, NSP14 enhances *de novo* guanosine nucleotide synthesis, elevating GTP levels that fuel m^7^GTP production [28]. This novel RNA modification mechanism reveals an unprecedented virus–host interaction strategy used by coronaviruses to reprogram host RNA metabolism. Interestingly, transcription-coupled incorporation of nucleotide analogs is widely exploited in both basic research and therapeutic applications. For example, 4-thiouridine (4-SU), 5-ethynyluridine (5-EU), and 5-bromouridine (5-BrU) are incorporated into nascent RNA transcripts and used to study RNA synthesis and turnover [73]. In cancer therapy, drugs such as 5-fluorouracil (5-FU) are incorporated into cellular RNA, interfering with RNA processing and function [74]. In antiviral therapies, nucleoside analogs such as remdesivir, favipiravir, ribavirin, and molnupiravir are incorporated into viral RNA by viral RNA-dependent RNA Pols. This incorporation leads to premature chain termination, lethal mutagenesis, or disruption of RNA synthesis, which ultimately inhibits viral replication [75–78]. Notably, ddhCTP, a naturally occurring antiviral nucleoside analog produced by the interferon-stimulated protein viperin, is also incorporated into viral RNA, where it halts viral RNA chain elongation [79]. These examples highlight how both viruses and host cells exploit RNA Pol-mediated incorporation of modified nucleotides as a strategy to modulate RNA function.

RNA modifications are key regulators of RNA structure and RNA–protein interactions [80]. Among these, m^6^A is well known for its role in weakening A–U base pairing through methylation at the *N*^6^ position of adenosine, which alters base-pairing dynamics at the Watson–Crick interface [81]. This structural destabilization exposes single-stranded regions that facilitate binding by proteins such as hnRNP-C and hnRNP-G [82–84], an effect termed the “m^6^A switch”. m^6^A also directly recruits m^6^A reader proteins such as the YTH family, influencing splicing, transcription, mRNA export, translation, and decay [80]. Consistently, depletion of METTL3, the key m^6^A methyltransferase, leads to splicing defects in hundreds of genes. Other modifications, such as *N*^1^-methyladenosine (m^1^A) and *N*^1^-methylguanosine (m^1^G), also impact RNA folding by disrupting non-canonical Hoogsteen base pairs, leading to A-form RNA destabilization [85]. Here, we found that NSP14 induces a dramatic increase in the internal mRNA m^7^G/G ratio, from 0.05% to 14%. m^7^G involves methylation at the *N*^7^ position of guanine nucleobase, introducing a positive charge without affecting Watson–Crick base pairing directly. However, this modification alters the electron distribution of guanosine, potentially disrupting non-canonical interactions. RNA G-quadruplexes (rG4s), structures stabilized by Hoogsteen hydrogen bonding between guanines, are particularly sensitive to m^7^G. rG4s play regulatory roles in pre-mRNA splicing, where they can enhance or suppress the use of nearby splice sites by recruiting specific RNA-binding proteins or remodeling local RNA structure [86]. Mimicking internal m^7^G modification with 7-deaza-deoxyguanosine (DAG) disrupts key hydrogen bonds, leading to rG4 destabilization [87]. By modulating rG4 stability, NSP14-induced internal m^7^G modifications may act as structural switches, analogous to the m^6^A switch, leading to AS.

An intriguing aspect of virus–host interactions is the potential evolutionary impact that viruses may have exerted on the architecture of host immune genes, particularly IFN-I. One of the defining features of mammalian and avian IFN-I genes is their intronless structure [88], with the notable exception of IFN-κ, which retains a single intron [89]. In contrast, ancestral IFN-I genes in fish and amphibians exhibit a multi-exon structure composed of five exons and four introns [90, 91]. It is hypothesized that the retroposition of intron-containing IFN-I transcripts gave rise to the intronless IFN-I genes found in amniotes, probably occurring during the evolutionary transition of vertebrates from aquatic to terrestrial environments [91]. This phylogenetic divergence raises an important question in evolutionary immunology: did selective pressure from pathogens favor the emergence of intronless IFN-I genes in amniotes? [92] Our findings that SARS-CoV-2 NSP14 promotes intron retention in host transcripts via internal m^7^G modification suggest that intron-containing mRNAs may be especially vulnerable to virus-induced post-transcriptional disruption. Molecular clock analyses suggest that the most recent common ancestor of coronaviruses could date back as far as 190–489 million years [93], potentially overlapping with early vertebrate or amniote evolution. Importantly, many other ancient virus families, such as rhabdoviruses and paramyxoviruses, have co-evolved with vertebrates for hundreds of millions of years and are known to interfere with host RNA processing and splicing. Given that IFN-I serves as a central antiviral effector, its intronless architecture may have evolved to ensure rapid, splicing-independent expression in response to viral immune invasion, escaping viral manipulation of RNA processing. Together, these observations support a model in which viruses that disrupt host RNA splicing may have contributed to the evolution of IFN-I genes, favoring intronless gene structures that are resistant to splicing-based antagonism.

## Limitations

Our study reveals a novel mechanism by which coronavirus NSP14 induces internal m^7^G modification and alters host mRNA splicing. Several limitations should be acknowledged. First, while we demonstrate that m^7^GTP incorporation is RNA Pol II dependent, the specific sequence or structural features that guide internal m^7^G placement remain unclear. Future studies using cross-linking or high-resolution mapping will be required to define the m^7^G consensus sites and their transcriptomic distribution. Second, although anti-m^7^G immunofluorescence and m^7^G FACS provide useful readouts of cellular m^7^G signal, both approaches rely on antibody-based detection and cannot definitively distinguish free m^7^G-containing metabolites, such as m^7^GTP, from RNA-incorporated m^7^G species. Therefore, these assays were used primarily as cellular readouts of global m^7^G changes. Third, although we show that altered mRNA splicing correlates with increased intron retention and impaired antiviral gene expression, the functional consequences of specific splice isoform changes, such as their stability, translation, or contribution to viral replication and immune evasion, were not evaluated. Fourth, although inhibition of NSP14-induced m^7^G modification and AS reduces viral replication, the precise downstream host pathways or genes driving this antiviral effect are still unclear. Functional studies linking specific splicing changes to viral fitness or immune evasion will be crucial to understanding the broader implications of NSP14 activity. Lastly, the potential interplay between NSP14-induced m^7^G modification and other epitranscriptomic marks (e.g., m^6^A and m^5^C) remains an open question that requires further investigation.

## Supplementary Material

gkag829_Supplemental_Files

## Data Availability

The RNA-seq data have been deposited in GEO under accession GSE307386. The molecular models have been provided as supplementary data.
