# Supplementary material for: Coronavirus NSP14 drives internal m7G modification to rewire host splicing and promote viral replication
Source: Nucleic Acids Res. 2026 Aug 21;54(15):gkag829. doi: 10.1093/nar/gkag829 (PMC13494556; doi:10.1093/nar/gkag829)
Supplement: Supplementary file 1 [file gkag829_supplemental_files.zip › 2025 Revision Supplementary v2.pdf]

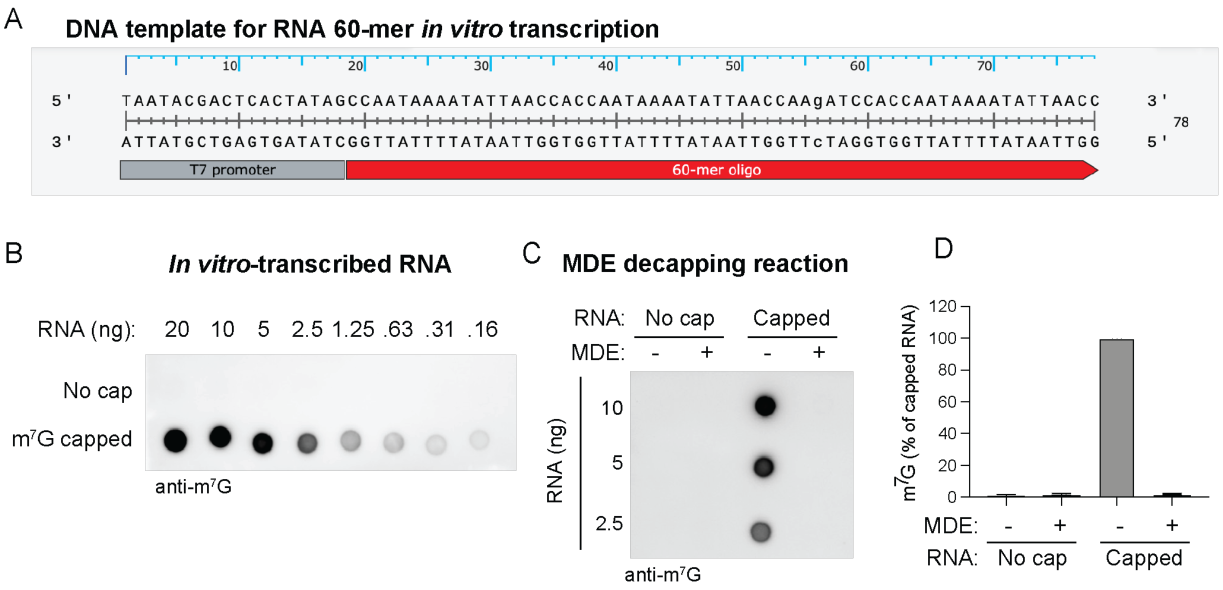

Figure S1. Messenger Decapping Enzyme efficiently removes 5' cap-derived m^{7}G signal from *in vitro*-transcribed RNA:

(A) Schematic of the DNA template used to generate a 60-nt *in vitro*-transcribed RNA. The template contains a T7 promoter followed by the 60-mer RNA sequence.

(B) Uncapped or m^{7}G-capped *in vitro*-transcribed RNA was synthesized using MEGAscript T7 Transcription Kit (Invitrogen, AM1334) in the absence or presence of 3'-O-Me-m7G(5')ppp(5')G RNA Cap Structure Analog (NEB, S1411S). Serially diluted RNA inputs were analyzed by anti-m^{7}G dot blot. m^{7}G-capped RNA produced a dose-dependent anti-m^{7}G signal.

(C) Uncapped or m^{7}G-capped *in vitro*-transcribed RNA was treated with or without Messenger Decapping Enzyme (MDE, M0608S) and analyzed by anti-m^{7}G dot blot. MDE treatment significantly reduced the m^{7}G signal from capped RNA.

(D) Densitometric quantification of the dot blot signal in (C). The m^{7}G signal was normalized to untreated capped RNA. MDE treatment removed the cap-derived m^{7}G signal with >99% efficiency. These results demonstrate that MDE efficiently removes 5′ cap-derived m^{7}G.

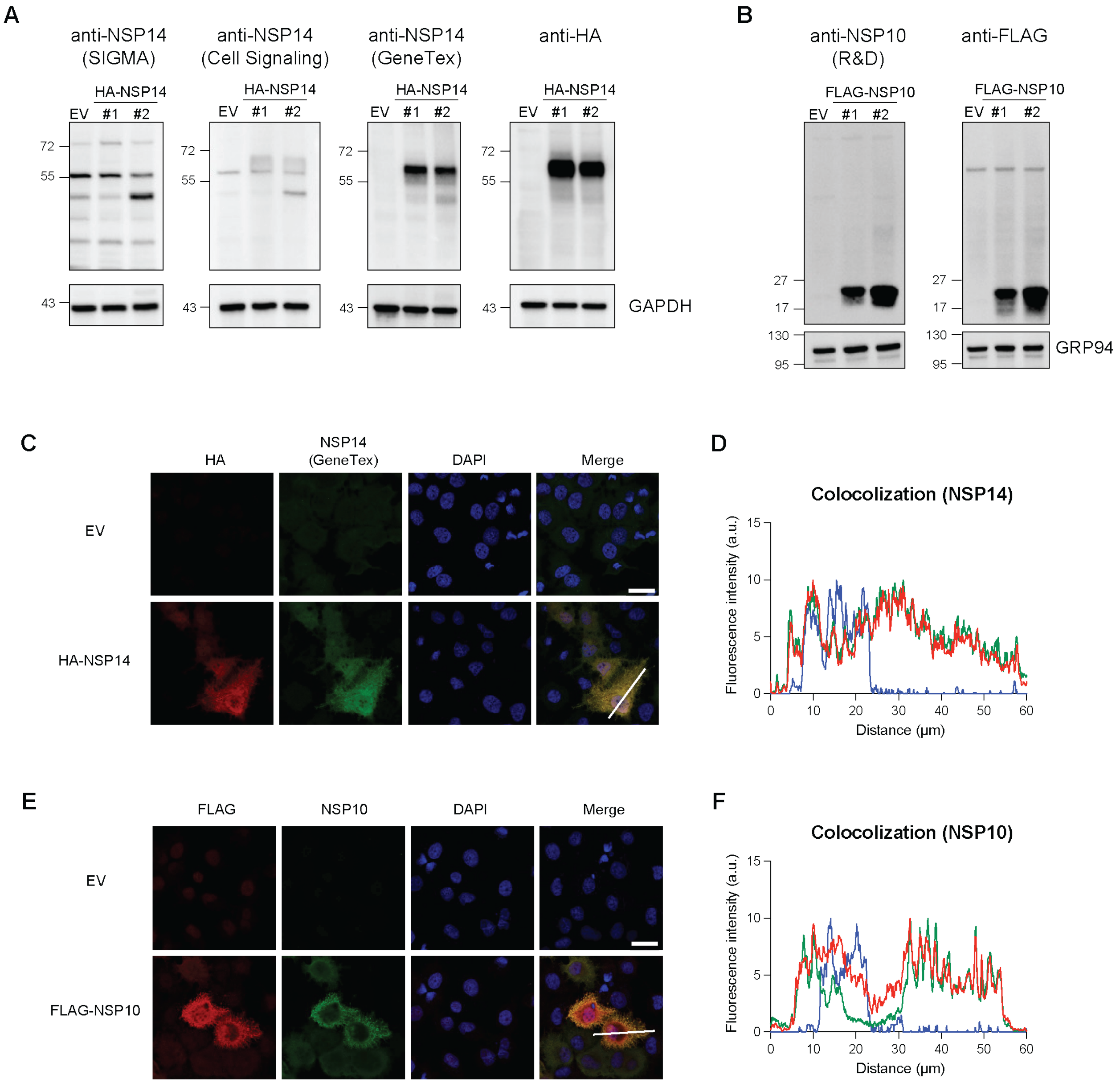

Figure S2. Validation of anti-NSP14 and anti-NSP10 antibodies for immunoblotting and immunofluorescence:

(A) Immunoblot analysis of HEK293T cells transfected with empty vector (EV) or HA-tagged SARS-CoV-2 NSP14. Cell lysates were probed with three commercial anti-NSP14 antibodies from Sigma (SAB3501144), Cell Signaling Technology (#99098) and GeneTex (HL2578), or with anti-HA antibody as a positive control for HA-NSP14 expression. GAPDH was used as a loading control. Among the tested antibodies, the GeneTex anti-NSP14 antibody detected a band corresponding to HA-NSP14 with a pattern similar to anti-HA.

(B) Immunoblot analysis of HEK293T cells transfected with EV or FLAG-tagged SARS-CoV-2 NSP10. Cell lysates were probed with anti-NSP10 antibody or anti-FLAG antibody as a positive control for FLAG-NSP10 expression. GRP94 was used as a loading control.

(C) Confocal immunofluorescence analysis of HeLa cells transfected with EV or HA-NSP14 and stained with anti-HA and the GeneTex anti-NSP14 antibody. DAPI was used to stain nuclei. The anti-NSP14 signal was detected in HA-NSP14-expressing cells and overlapped with the HA signal. Scale bar, 25 μm.

(D) Line-scan fluorescence intensity analysis across the indicated region in (C), showing colocalization between anti-HA and anti-NSP14 signals.

(E) Confocal immunofluorescence analysis of HeLa cells transfected with EV or FLAG-NSP10 and stained with anti-FLAG and anti-NSP10 antibodies. DAPI was used to stain nuclei. The anti-NSP10 signal was detected in FLAG-NSP10-expressing cells and overlapped with the FLAG signal. Scale bar, 25 μm.

(F) Line-scan fluorescence intensity analysis across the indicated region in (E), showing colocalization between anti-FLAG and anti-NSP10 signals. Together, these results support the use of the GeneTex anti-NSP14 and anti-NSP10 antibodies to identify NSP14- and NSP10-positive cells by immunoblotting and immunofluorescence.

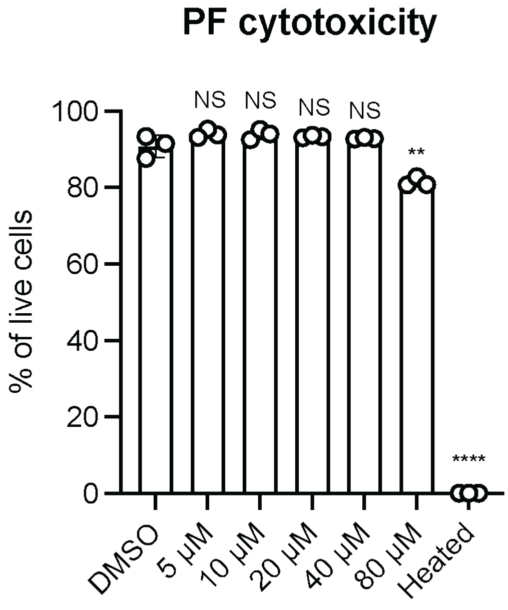

Figure S3. Cytotoxicity assay of PF-03882845:
HEK293T cells were treated with increasing concentrations of PF-03882845 (PF) for 24 h. Cell viability was assessed using a cytotoxicity assay kit (Invitrogen; L34976) following the manufacturer's protocol and analyzed by flow cytometry. Data are presented as mean ± SD from three biological replicates. **P < 0.01, ****P < 0.001 by unpaired Student's t-test. NS, not significant.

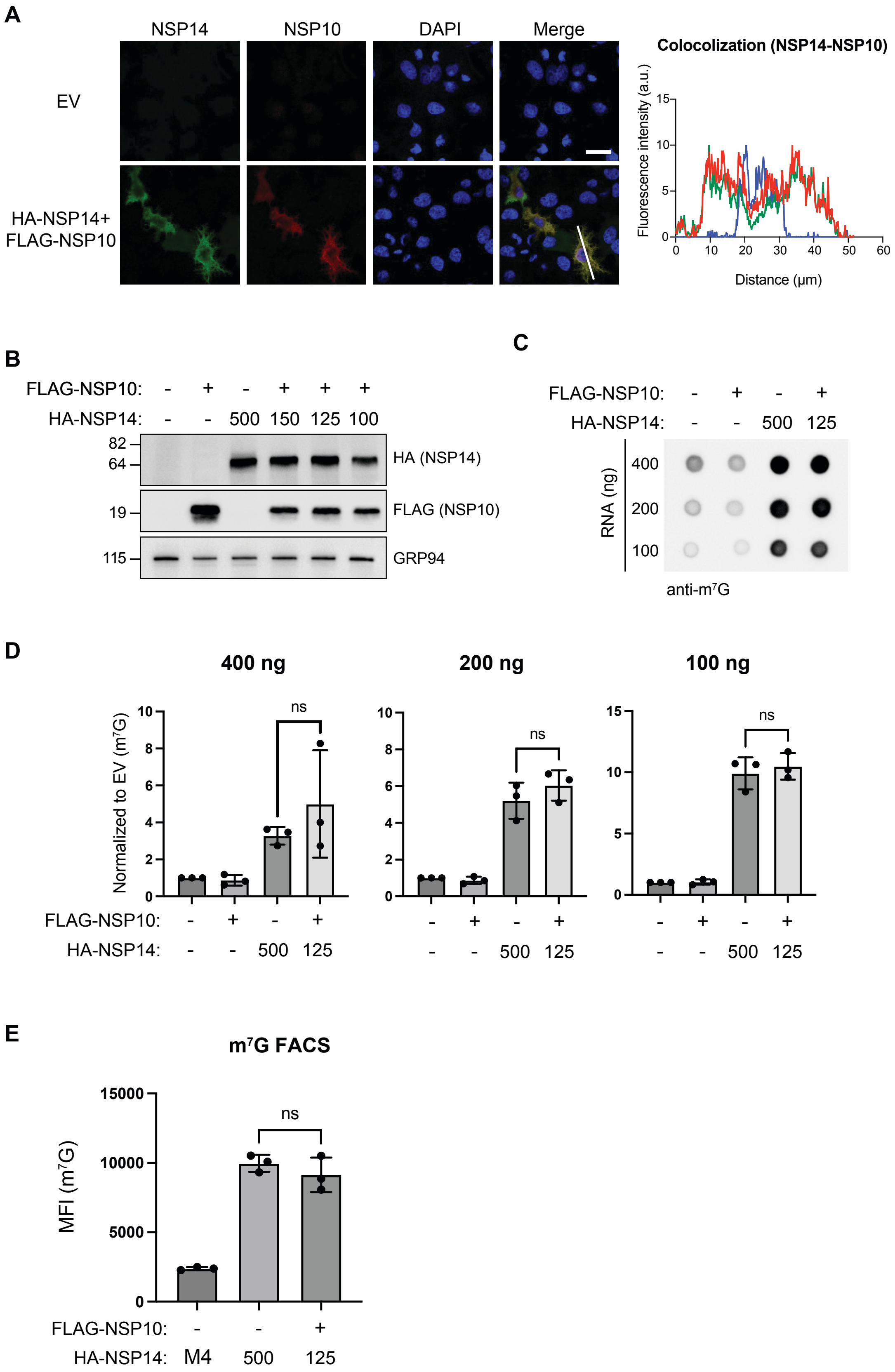
A
NSP14
NSP10
DAPI
Merge
EV
HA-NSP14+
FLAG-NSP10
Colocolization (NSP14-NSP10)
Fluorescence intensity (a.u.)
Distance (μm)
B
FLAG-NSP10: - + - + + +
HA-NSP14: - - 500 150 125 100
82
64
19
115
HA (NSP14)
FLAG (NSP10)
GRP94
C
FLAG-NSP10: - + - +
HA-NSP14: - - 500 125
RNA (ng)
400
200
100
anti-m7G
D
400 ng
200 ng
100 ng
Normalized to EV (m7G)
ns
FLAG-NSP10: - + - +
HA-NSP14: - - 500 125
E
m7G FACS
MFI (m7G)
ns
FLAG-NSP10: - - +
HA-NSP14: M4 500 125

Figure S4. NSP10 increases NSP14-induced m^{7}G modification primarily by increasing NSP14 protein abundance:
(A) Confocal immunofluorescence analysis of HeLa cells transfected with empty vector (EV) or co-transfected with plasmids encoding HA-tagged NSP14 (HA-NSP14) and FLAG-tagged NSP10 (FLAG-NSP10). Cells were stained with anti-NSP14 and anti-NSP10 antibodies, and nuclei were counterstained with DAPI. NSP14 and NSP10 signals showed overlapping cytoplasmic/perinuclear staining in co-transfected cells. Right: The line-scan fluorescence intensity plot shows signal overlap across the indicated region. Scale bar, 25 μm.

(B) Immunoblot analysis of HEK293T cells transfected with plasmids encoding HA-tagged NSP14 (HA-NSP14) alone or co-transfected with FLAG-tagged NSP10 (FLAG-NSP10) for 24 h. A fixed amount of FLAG-NSP10 was used, and HA-NSP14 was titrated (100, 125 and 150 ng). Empty vector was added to normalize the total plasmid input. Cell lysates were subjected to immunoblot analysis with anti-HA, anti-FLAG and anti-GRP94 antibodies.

(C) RNA m^{7}G immunoblot analysis of HEK293T cells transfected with the indicated plasmids. After 24 h, total RNA was TRIzol-extracted and subjected to m^{7}G immunoblotting and methylene blue staining.

(D) Quantification of m^{7}G immunoblot signals from three biological replicates using the indicated RNA inputs. m^{7}G signals were normalized to the EV control.

(E) Flow cytometry-based m^{7}G analysis of HEK293T cells transfected with the indicated plasmids. Cells were fixed, stained with anti-m^{7}G and anti-HA antibodies and analyzed by flow cytometry.

Data are presented as mean ± SD from three biological replicates. NS, not significant by unpaired Student's t test.

A

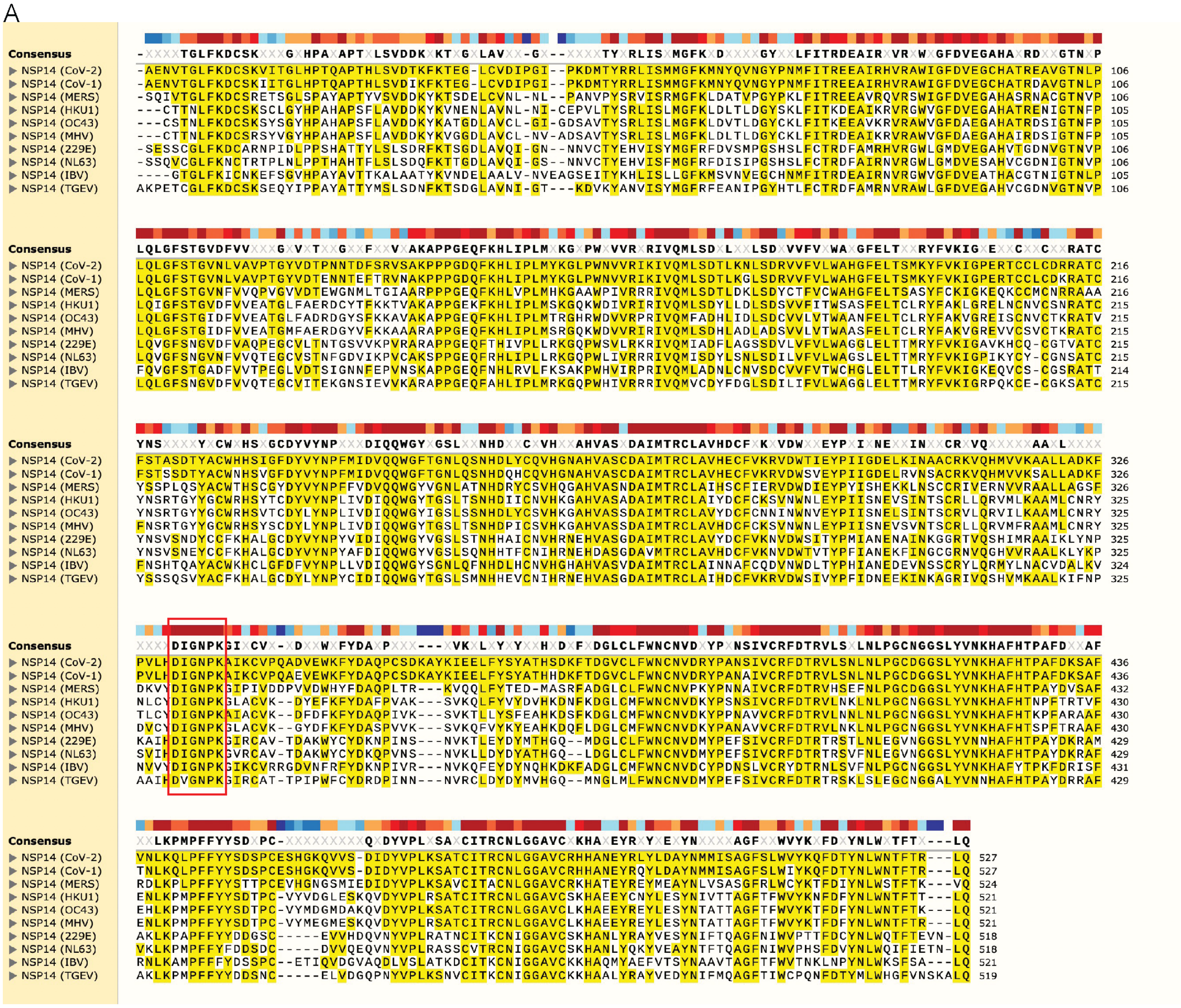

B

Virus	Genus	Host	Protein seq
HCoV-229E	Alphacoronavirus	Human	NC_002645
HCoV-NL63	Alphacoronavirus	Human	NC_005831
HCoV-HKU1	Betacoronavirus	Human	NC_006577
HCoV-OC43	Betacoronavirus	Human	NC_006213
MERS-CoV	Betacoronavirus	Human	NC_019843
SARS-CoV-1	Betacoronavirus	Human	NC_004718
SARS-CoV-2	Betacoronavirus	Human	NC_045512.2

C

Similarity (%)	HCoV-229E	HCoV-NL63	HCoV-HKU1	HCoV-OC43	MERS-CoV	SARS-CoV-1	SARS-CoV-2
HCoV-229E	X						
HCoV-NL63	89	X					
HCoV-HKU1	68	68	X				
HCoV-OC43	67	68	91	X			
MERS-CoV	66	68	74	74	X		
SARS-CoV-1	68	67	74	75	77	X	
SARS-CoV-2	68	67	74	74	77	99	X

Figure S5. Sequence analysis of coronavirus NSP14 proteins:
(A) Protein sequence alignment of NSP14 from representative coronaviruses. Conserved amino acid residues are highlighted in yellow, and the red box indicates the S-adenosylmethionine (SAM) binding motif. The alignment includes alphacoronaviruses (HCoV-229E [229E], HCoV-NL63 [NL63], transmissible gastroenteritis virus [TGEV]), betacoronaviruses (HCoV-HKU1 [HKU1], HCoV-OC43 [OC43], MERS-CoV [MERS], SARS-CoV [CoV-1], SARS-CoV-2 [CoV-2], mouse hepatitis virus [MHV]) and the gammacoronavirus infectious bronchitis virus (IBV).

(B-C) NSP14 sequence identity among human coronaviruses. Heatmap showing pairwise amino acid sequence similarity (%) of NSP14 across human coronaviruses.

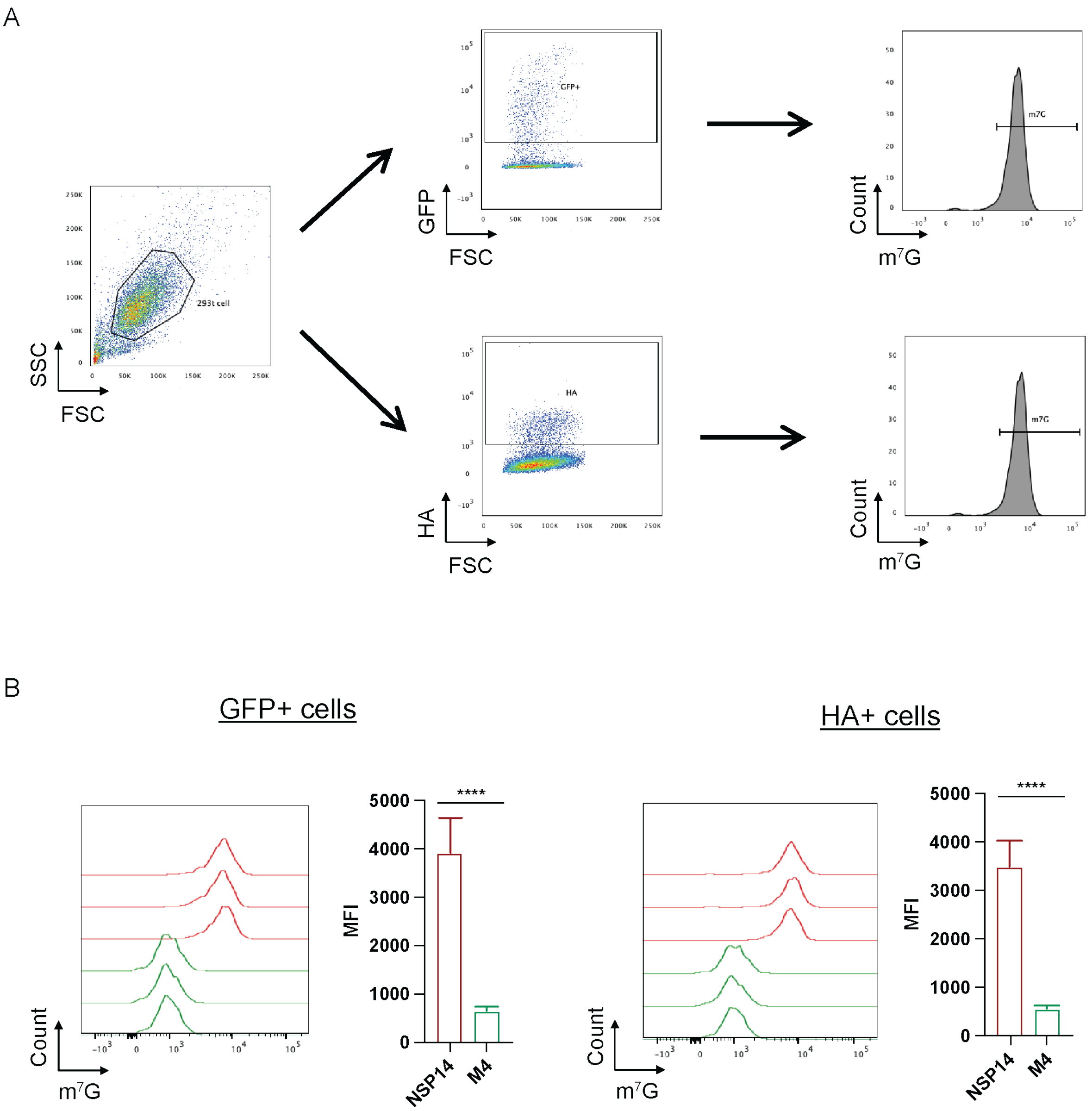

Figure S6. Flow cytometry-based detection of RNA m^{7}G modification (m^{7}G FACS): (A) Schematic overview of the flow cytometry strategy used to detect m^{7}G modification. HEK293T cells were transfected with plasmids encoding HA- and GFP-tagged NSP14 for 24 h. Cells were fixed, permeabilized and stained with antibodies against m^{7}G and HA, followed by flow cytometry analysis.

(B) Flow cytometry histograms and quantification of mean fluorescence intensity (MFI) showing m^{7}G signals in GFP$^+$ or HA$^+$ cells transfected with NSP14 or the *N7*-MTase mutant (M4). NSP14 significantly increased m^{7}G levels compared to the M4 mutant. Data are shown as mean ± SD from three biological replicates. ****P < 0.0001 by unpaired Student's t test.

A

PDB ID	Species	Complex	Ligand(s)
9VCL	SARS-CoV-2	NSP10 and RNA	ATMP
9VCK	SARS-CoV-2	NSP10 and RNA	SMP
8R7B	SARS-CoV-2	-	SAH and TDI-015051
7R2V	SARS-CoV-2	-	SAH
7QIF	SARS-CoV-2	-	7MeGpppG
7N0B	SARS-CoV-2	NSP10 and RNA	-
5C8U	SARS-CoV	NSP10	-
5C8T	SARS-CoV	NSP10	SAM
5C8S	SARS-CoV	NSP10	SAH and GpppA
5SM0	SARS-CoV-2	-	Z32665176

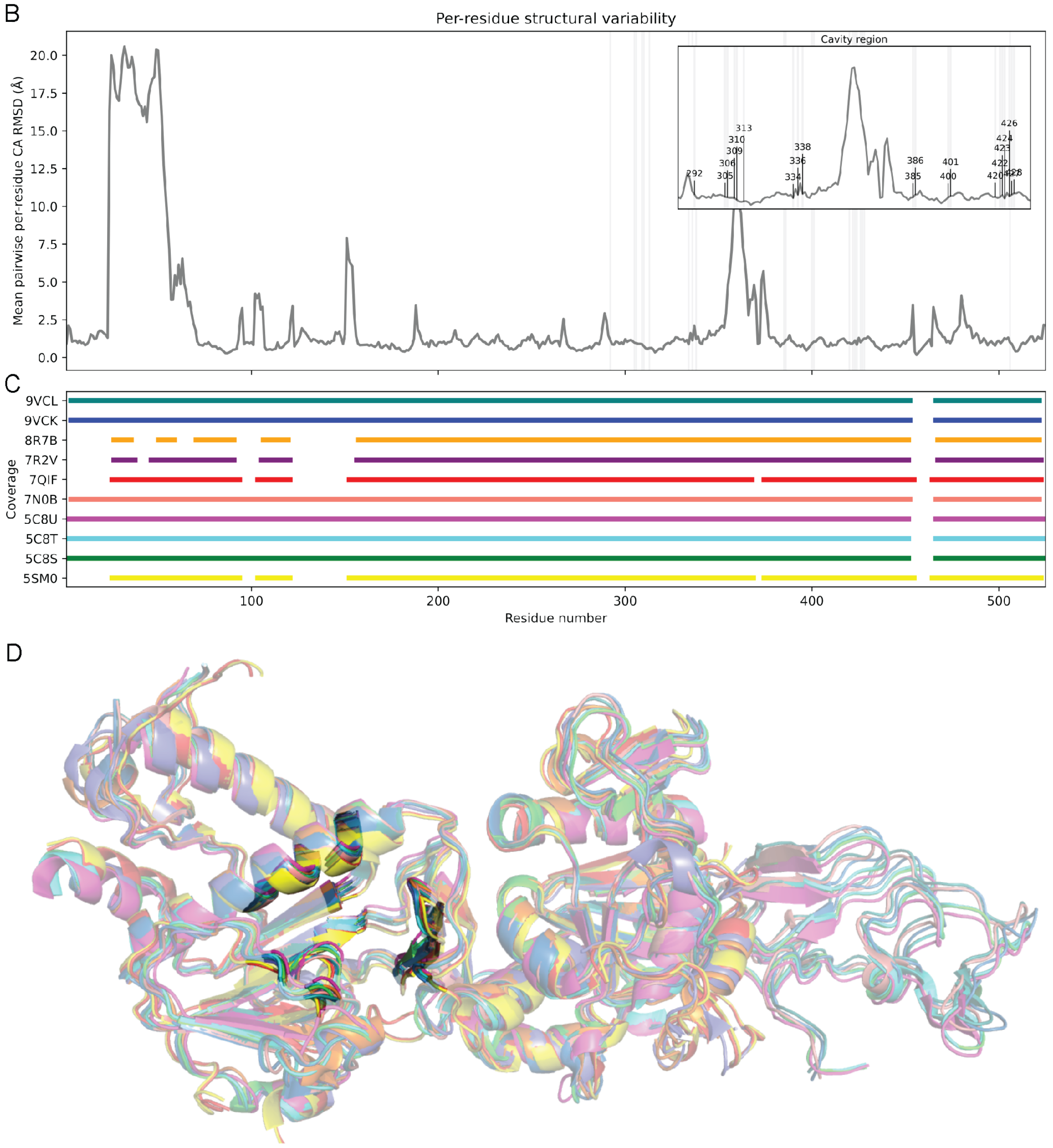

Figure S7. Structural conservation of the NSP14 nucleotide-binding cavity:
(A) List of NSP14 structures used for structural comparison, including SARS-CoV and SARS-CoV-2 NSP14 structures with or without NSP10, RNA, SAM/SAH or nucleotide/cap analog ligands.

(B) Per-residue structural variability analysis of aligned NSP14 structures. Mean pairwise Cα RMSD values are shown across NSP14 residues, with the inset highlighting residues within the nucleotide-binding cavity.

(C) Structural coverage map of the aligned NSP14 structures shown in (A). Regions with incomplete coverage indicate flexible or unresolved segments.

(D) Superposition of the NSP14 structures listed in (A). The analysis shows that, despite structural variability in other regions, the nucleotide-binding cavity (highlighted) is structurally conserved across available NSP14 structures, supporting the use of this well-defined cavity geometry for molecular docking.

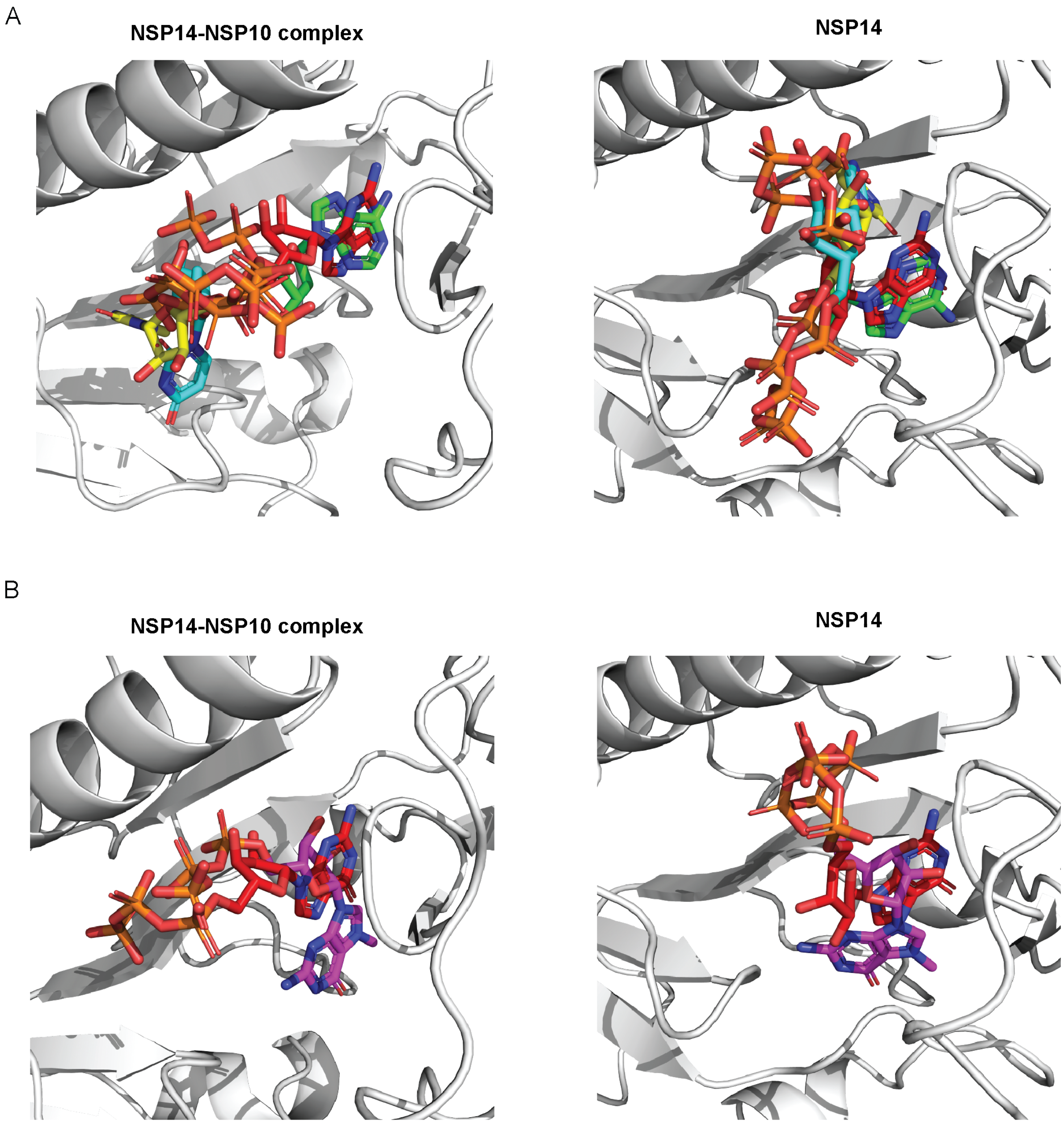

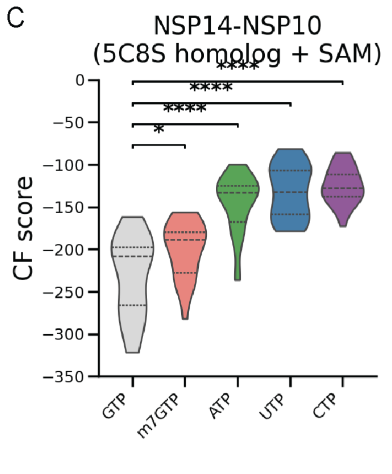

D

Target	Ligand	Distance
5C8S (complex)	GpppA (Imidazole)	3.64 Å
5C8S (complex)	GpppA (Pyrimidine)	4.50 Å
7QIF (complex)	7MeGpppG (Imidazole)	4.34 Å
7QIF (complex)	7MeGpppG (Pyrimidine)	4.55 Å
5C8S homolog (docking)	GTP (Imidazole)	3.41 Å
5C8S homolog (docking)	GTP (Pyrimidine)	4.71 Å
5C8S homolog (docking)	m7GTP (Imidazole)	4.87 Å
5C8S homolog (docking)	m7GTP (Pyrimidine)	4.94 Å
5C8S homolog (docking)	GTP (Imidazole) + SAM	4.98 Å
5C8S homolog (docking)	GTP (Pyrimidine) + SAM	3.71 Å
5C8S homolog (docking)	m7GTP (Imidazole) + SAM	3.90 Å
5C8S homolog (docking)	m7GTP (Pyrimidine) + SAM	4.64 Å

Figure S8. Molecular docking analysis of nucleotide triphosphate binding to the NSP14-NSP10 complex and NSP14:
Structural modeling and docking were performed to assess the binding of nucleotide triphosphates to the NSP14-NSP10 complex and NSP14. Docking poses were generated using a homology model based on the SARS-CoV-1 NSP14-NSP10 complex (PDB 5C8S) and the SARS-CoV-2 NSP14 structure bound to the cap analog m7GpppG (PDB 7QIF). (A) Docking poses of GTP (red), ATP (green), UTP (cyan) and CTP (yellow) indicate that purine-based NTPs (GTP and ATP) align within the binding cavity, while pyrimidine-based NTPs (UTP and CTP) are displaced, suggesting reduced binding compatibility.

(B) Comparison of docking poses of GTP (red) and m^7GTP (magenta) shows that the *N*7-methylated guanine of m^7GTP extends outside the binding cavity, consistent with its role as a reaction product rather than a substrate.

(C) Docking analysis of NTP binding to the NSP14-NSP10 model with SAM included as a fixed molecule in the active site. The presence of SAM produced similar relative NTP scoring, supporting the predicted preference for GTP when SAM is included in the model. Data are shown as violin plots representing the top five poses from five independent docking runs (25 poses in total). *P < 0.05, ****P < 0.001 by unpaired Student’s t test.

(D) Summary of centroid-to-centroid distances between the guanine base and the F426 aromatic ring in experimentally solved NSP14 complexes and docking models. Distances are shown for the imidazole and pyrimidine rings of the guanine base and are consistent with a potential π–π stacking interaction.

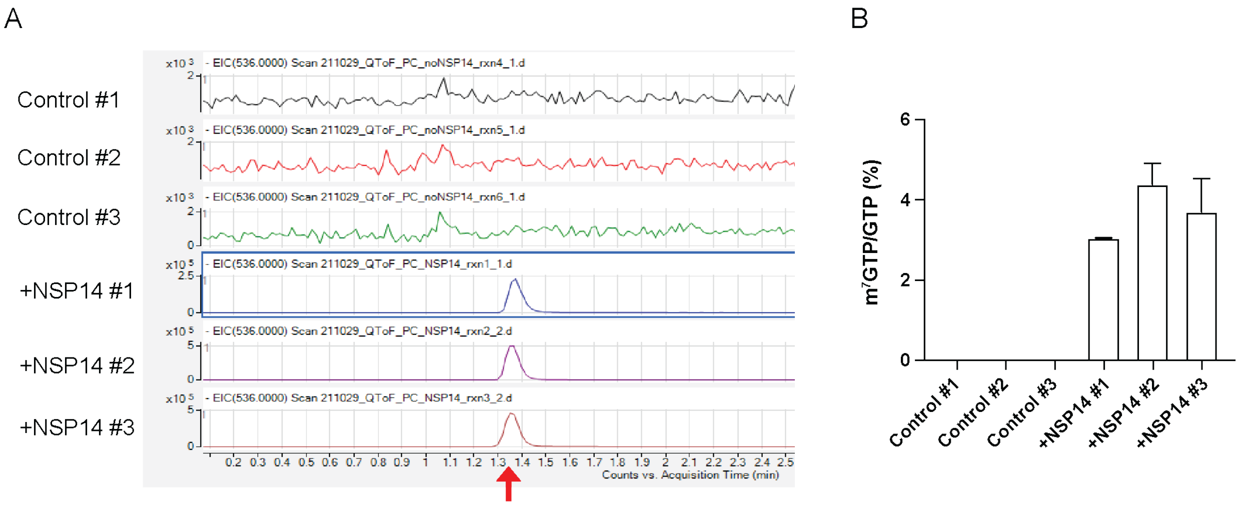

Figure S9. Mass spectrometry confirms NSP14-mediated synthesis of m^{7}GTP:
(A) Extracted ion chromatograms from liquid chromatography-quadrupole time-of-flight (LC-QToF) mass spectrometry showing detection of m^{7}GTP in *in vitro* methylation reactions with or without recombinant NSP14-NSP10 complex. A distinct peak corresponding to m^{7}GTP (red arrow) was detected in all NSP14-treated samples but absent in control reactions.

(B) Quantification of m^{7}GTP as a percentage of total GTP from three biological replicates. Data are presented as mean ± SD.

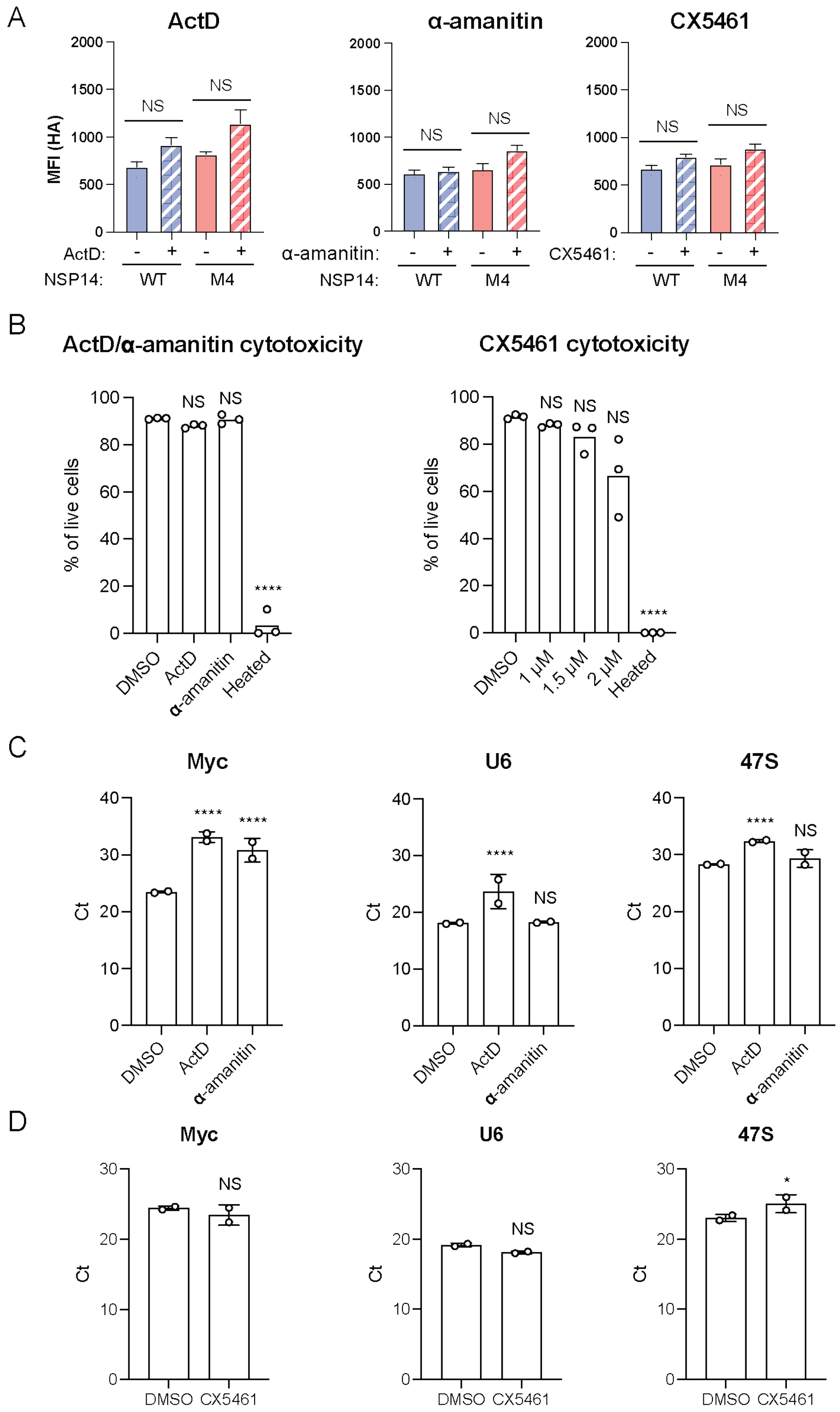

Figure S10. NSP14 expression, cytotoxicity and validation of transcription inhibitor activity:

(A) Flow cytometry analysis of WT NSP14 and M4 expression in HEK293T cells treated with transcription inhibitors. Cells were transfected with *in vitro*-transcribed RNA encoding WT NSP14 or M4 and treated with actinomycin D (ActD), α-amanitin or CX-5461 for 24 h. NSP14 and M4 protein expression was quantified by anti-HA staining from the same experiments used to measure m^7G levels in Figure 7.

(B) HEK293T cells were treated with actinomycin D, α-amanitin or CX-5461 for 24 h. Cell viability was assessed using a cytotoxicity assay kit (Invitrogen; L34976) following the manufacturer's protocol and analyzed by flow cytometry.

(C, D) RNA levels of the indicated genes in HEK293T cells treated with transcription inhibitors were determined by RT-qPCR. Myc, U6 and 47S were used as representative transcripts for RNA Pol II, Pol III and Pol I activity, respectively.

Data are presented as mean ± SD from two to three biological replicates. *P < 0.05, ****P < 0.001 by unpaired Student's t test. NS, not significant.

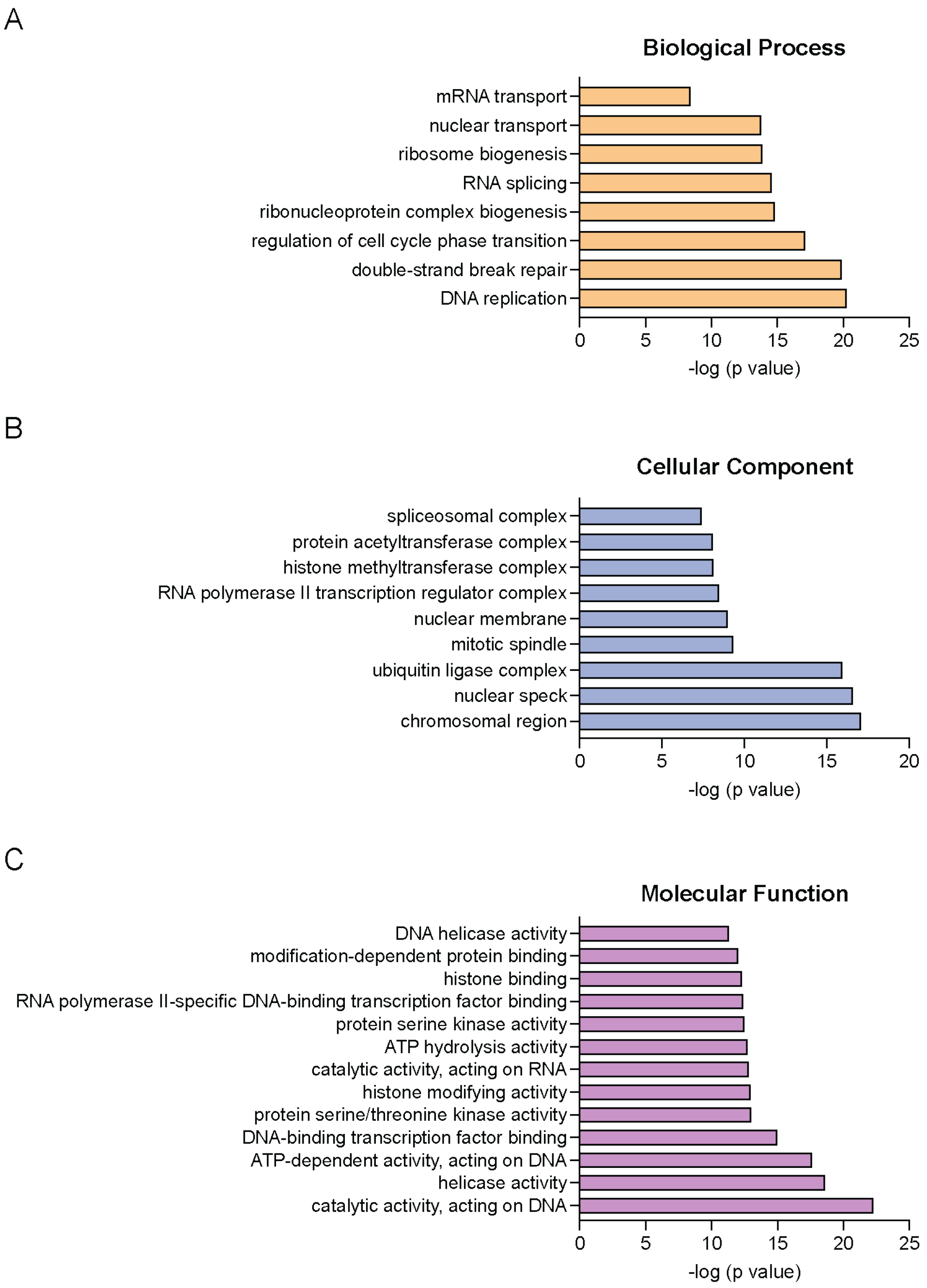

Figure S11. Gene ontology (GO) enrichment analysis of genes with increased intron retention upon NSP14 expression:
(A) Biological Process terms show enrichment for pathways involved in genome maintenance and RNA metabolism, including DNA replication/repair, cell cycle regulation and RNA processing.

(B) Cellular Component terms highlight enrichment in nuclear structures such as chromosomal regions, spliceosomal complexes and nuclear specks, consistent with roles in chromatin organization and RNA processing.

(C) Molecular Function terms are enriched for DNA- and RNA-related enzymatic activities, helicases, transcription factor binding and chromatin-modifying enzymes. Together, these results indicate that NSP14 disrupts host pathways coordinating genome stability, RNA splicing and transcriptional regulation.

A

Gene	Coordination	Intron size (bp)	Exons	dPSI	IR	RT-PCR amplicon size Long (bp)	RT-PCR amplicon size Short (bp)
PRRC2A	chr6:31637325-31637445	121	30 and 31	36.54	UP	204	83
ATG101	chr12:52070243-52070353	111	1 and 2	35.84	UP	201	90
RPS27A	chr2:55232709-55232807	99	1 and 2	29.78	UP	185	86
MAFG	chr17:81923058-81923149	92	2 and 3	37.92	UP	200	108
SSBP3	chr1:54228357-54228448	92	16 and 17	-0.44	Control	199	107

B

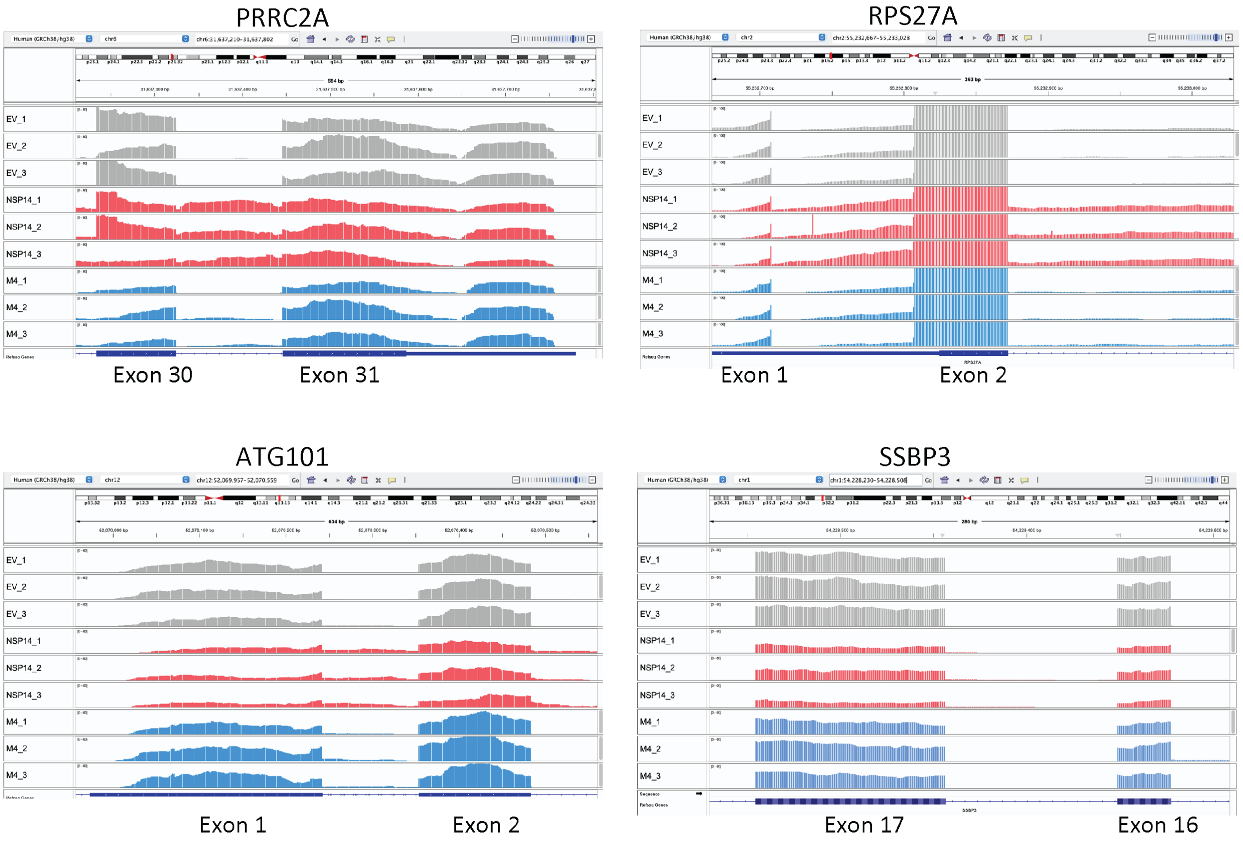

Figure S12. RNA-Seq analysis of NSP14-induced intron retention:
(A) Selected genes analyzed for intron retention, including genomic coordinates, intron size, involved exons, difference in percent spliced-in index (dPSI), intron retention (IR) status and RT-PCR amplicon sizes. PRRC2A, ATG101 and RPS27A displayed increased intron retention upon NSP14 expression, while SSBP3 served as a control with no significant changes. Data extracted from Zaffagni *et al.*, 2022.

(B) Representative IGV alignment tracks of PRRC2A, RPS27A, ATG101 and SSBP3 showing intron retention. RNA-seq read coverage is displayed for cells transfected with empty vector (EV, gray), wild-type NSP14 (red) and the M4 mutant (blue). Increased intron retention is observed in PRRC2A, RPS27A and ATG101 upon NSP14 expression, while SSBP3 shows no change and serves as a negative control.

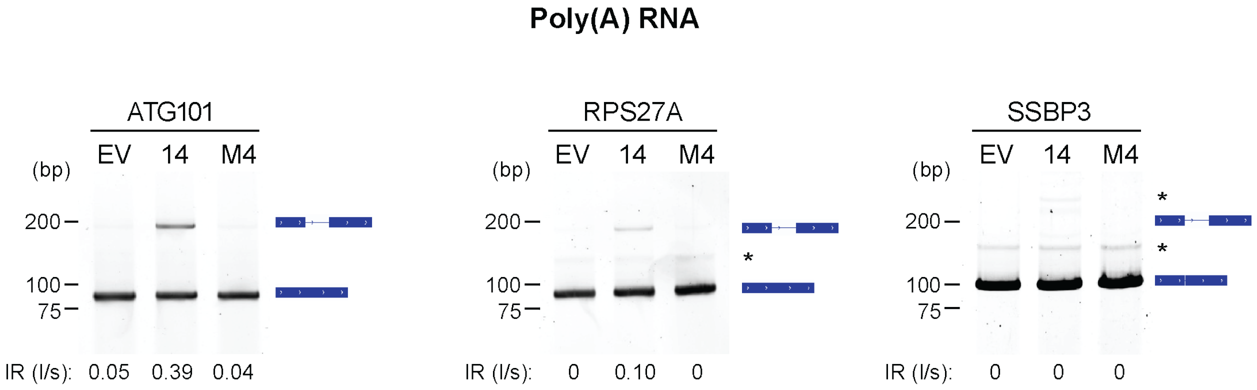

Figure S13. RT-PCR analysis of intron retention in poly(A) RNA:
HEK293T cells were transfected with EV, NSP14 or M4 for 24 h. Poly(A) RNA was reverse-transcribed and amplified by PCR. The resulting PCR products were analyzed by DNA gel electrophoresis. The intron retention ratio of unspliced "long" fragments to spliced "short" fragments (IR(l/s)) was quantified using ImageJ. *, nonspecific PCR product.

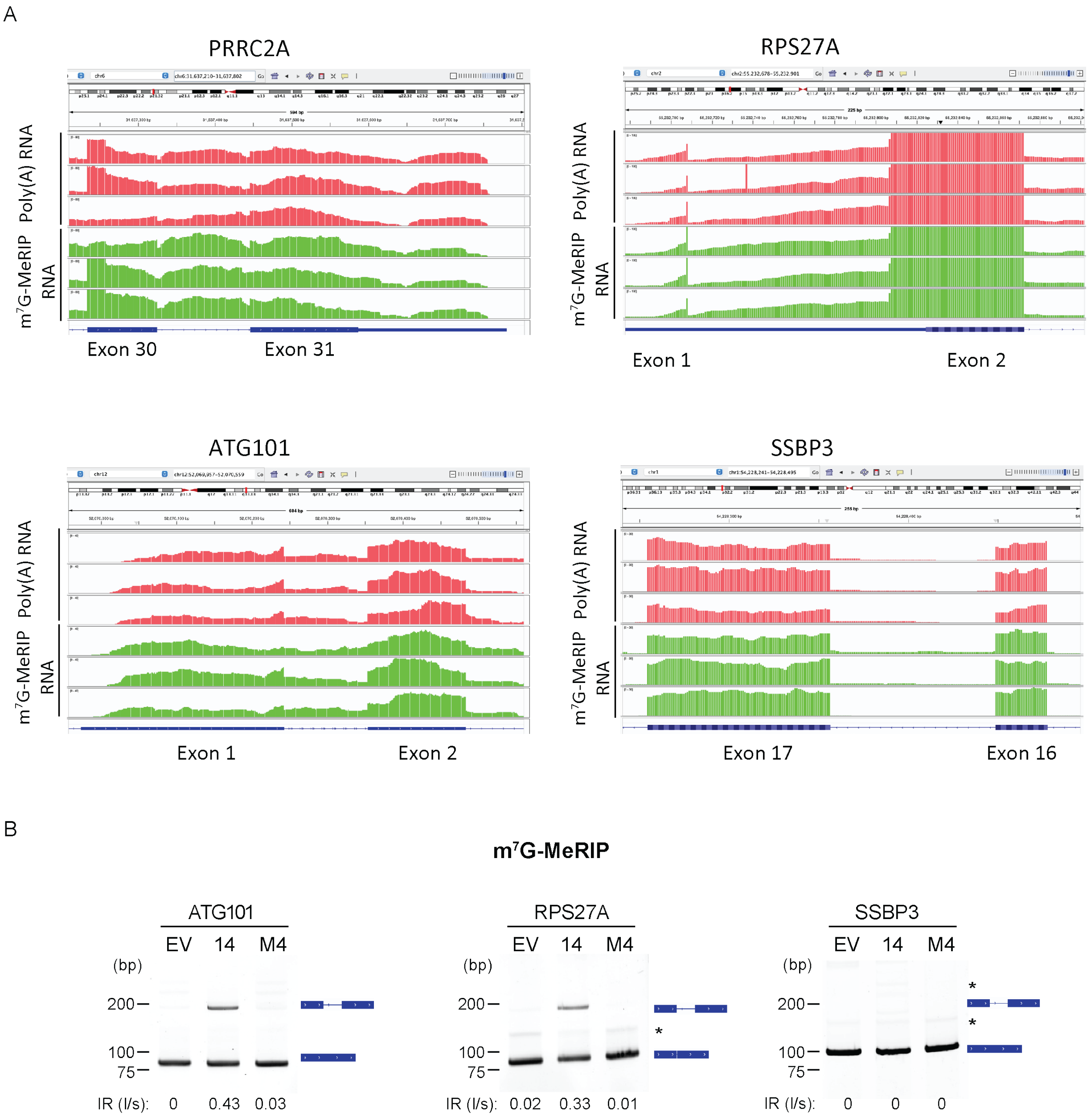

Figure S14. NSP14-induced intron retention is enriched in m^{7}G-modified RNA: (A) Representative IGV alignment tracks of PRRC2A, RPS27A, ATG101 and SSBP3, comparing m^{7}G-MeRIP RNA and poly(A) RNA. Read coverage is shown for cells transfected with empty vector (EV, gray), NSP14 (red) and the M4 mutant (blue). Notably, increased intron retention is observed in the m^{7}G-MeRIP RNA compared to the poly(A) RNA.

(B) Validation of NSP14-induced intron retention by RT-PCR from m^{7}G-MeRIP RNA. HEK293T cells were transfected with EV, NSP14 or M4 mutant for 24 h. Purified m^{7}G-modified RNA was reverse-transcribed and amplified with primers flanking retained introns. The resulting PCR products were analyzed by DNA gel electrophoresis. The

intron retention ratio of unspliced “long” fragments to spliced “short” fragments (IR(l/s)) was quantified using ImageJ. *, non-specific PCR product.

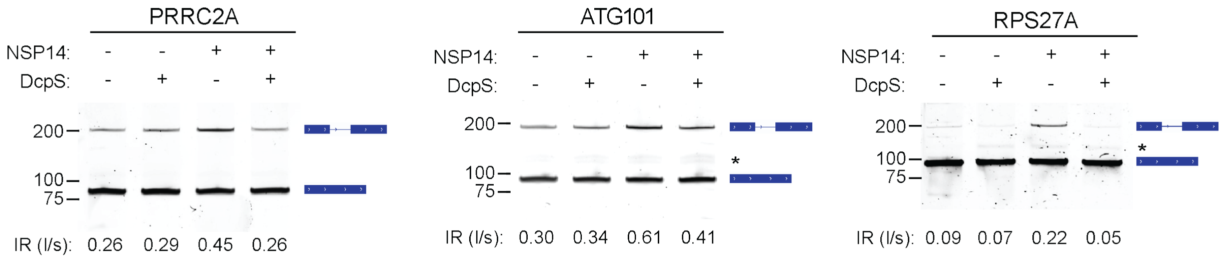

Figure S15. DcpS reduces NSP14-induced intron retention in total RNA: HEK293T cells were transfected with EV or NSP14 in the absence or presence of DcpS for 24 h. Total RNA was reverse-transcribed and amplified by PCR. The resulting PCR products were analyzed by DNA gel electrophoresis. The intron retention ratio of unspliced "long" fragments to spliced "short" fragments (IR(l/s)) was quantified using ImageJ. Co-expression of DcpS reduced NSP14-induced intron retention, supporting a role for m^7GTP-dependent m^7G incorporation in NSP14-mediated splicing changes. *, nonspecific PCR product.

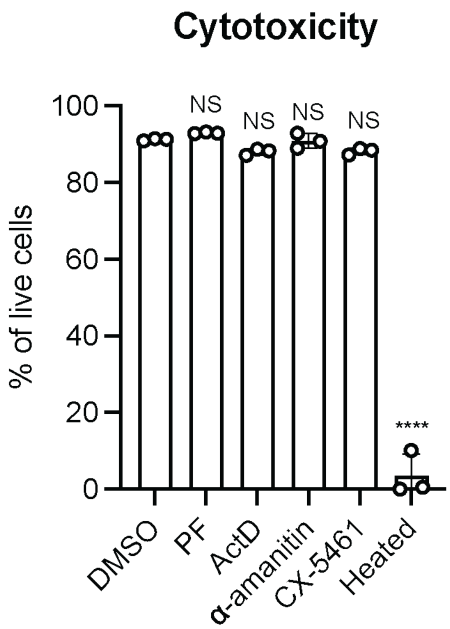

Figure S16. Cytotoxicity assay of inhibitors in Vero E6:
Vero E6 cells were treated with PF-03882845 (PF) and transcription inhibitors (actinomycin D [ActD], α-amanitin, CX-5461) for 24 h. Cell viability was assessed using a cytotoxicity assay kit (Invitrogen; L34976) following the manufacturer's protocol and analyzed by flow cytometry. Data are presented as mean ± SD from three biological replicates. ****P < 0.001 by unpaired Student's t test. NS, not significant.
